# Runx3 prevents spontaneous colitis by directing the differentiation of anti-inflammatory mononuclear phagocytes

**DOI:** 10.1371/journal.pone.0233044

**Published:** 2020-05-26

**Authors:** Shay Hantisteanu, Yosef Dicken, Varda Negreanu, Dalia Goldenberg, Ori Brenner, Dena Leshkowitz, Joseph Lotem, Ditsa Levanon, Yoram Groner

**Affiliations:** 1 Department of Molecular Genetics, The Weizmann Institute of Science, Rehovot, Israel; 2 Veterinary Resources, The Weizmann Institute of Science, Rehovot, Israel; 3 Bioinformatics Unit, The Weizmann Institute of Science, Rehovot, Israel; Toho University Graduate School of Medicine, JAPAN

## Abstract

Mice deficient in the transcription factor Runx3 develop a multitude of immune system defects, including early onset colitis. This paper demonstrates that Runx3 is expressed in colonic mononuclear phagocytes (MNP), including resident macrophages (RM) and dendritic cell subsets (cDC2). Runx3 deletion in MNP causes early onset colitis due to their impaired maturation. Mechanistically, the resulting MNP subset imbalance leads to up-regulation of pro-inflammatory genes as occurs in IL10R-deficient RM. In addition, RM and cDC2 display a marked decrease in expression of anti-inflammatory/TGF β-regulated genes and β-catenin signaling associated genes, respectively. MNP transcriptome and ChIP-seq data analysis suggest that a significant fraction of genes affected by Runx3 loss are direct Runx3 targets. Collectively, Runx3 imposes intestinal immune tolerance by regulating maturation of colonic anti-inflammatory MNP, befitting the identification of RUNX3 as a genome-wide associated risk gene for various immune-related diseases in humans, including gastrointestinal tract diseases such as Crohn’s disease and celiac.

## Introduction

RUNX3 is one of the three mammalian Runt-domain transcription factors (TFs) that are key gene expression regulators during development [1, 2]. *Runx3* was originally cloned based on its similarity to *Runx1* [3] and subsequently localized on human and mouse chromosomes 1 and 4, respectively [3, 4]. *Runx3*^-/-^ mice phenotypes reflect its expression pattern and necessity for the proper functioning of several important organs [2, 5]. Absence of Runx3 is associated with a multitude of defects in both adaptive and innate immunity arms, including: defective proliferation and differentiation of activated cytotoxic CD8^+^ T cells [6–9]; impaired induction of type 1 T-helper (Th1) cells [10] and activation of natural killer (NK) cells [11]; impaired development of intestinal innate lymphoid cells type 3 (ILC3) [12]; and lack of dendritic epithelial T cells [13]. We have also reported that Runx3 plays a pivotal role in MNP homeostasis. For example, Runx3 facilitates specification of splenic CD11b^+^ dendritic cells (DC) [14] and is required for development of TGFβ-dependent Langerhans cells in the skin [15]. Runx3^-/-^ bone marrow-derived DC (BMDC) are hyper-activated owing to deregulation of TGF-β-mediated maturation. Immune deficiencies in Runx3^-/-^ mice were also reported to cause lung inflammation associated with accumulation of hyper-activated DCs, leading to development of major hallmarks of asthma [15, 16]. Furthermore, Runx3^-/-^ mice spontaneously develop colitis at 4–6 weeks of age [17]. This disease is known to be closely associated with an impaired immune system. Thus it is interesting to note that human genome-wide association studies have linked RUNX3 to various immune-related diseases including asthma, ankylosing spondylitis, psoriasis, psoriatic arthritis, atopic dermatitis and gastrointestinal tract (GIT) diseases such as celiac and Crohn’s disease [2].

Expression of Runx3 in the GIT of wild type (WT) mice is confined to leukocytes and is not detected in GIT epithelial cells, indicating that cell-autonomous expression of Runx3 in leukocytes is involved in GIT homeostasis [18]. In view of the defects in different cell types of innate and adaptive immune systems in Runx3^-/-^ mice, and the association of RUNX3 with immune-related human diseases [2], we sought to determine which of the Runx3^-/-^ immune cell types is directly involved in colitis development. Previous research has shown that conditional deletion of Runx3 in NK cells and ILCs by crossing *Runx3*^*fl/fl*^ mice on to *Nkp46-Cre* mice does not induce spontaneous colitis, although those mice did show more severe intestinal damage following infection with *Citrobacter rodentium* [12]. Additionally, we have shown that *Runx3*^*-/-*^ mice are highly resistant to inflammation-dependent skin chemical carcinogenesis and this resistance is fully recapitulated in Runx3 conditional knockout mice, in which Runx3 was deleted in both DC and T cells, but not in epithelial cells [19].

Here, we show that conditional deletion of Runx3 specifically in MNP, but not in T cells, recapitulates the spontaneous colitis seen in *Runx3*^*-/-*^ mice. Specifically, Runx3 function in MNP is crucial for intestinal immune tolerance, as it regulates the proper maturation and anti-inflammatory functions of MNP.

## Materials and methods

### Mice

Mice lacking Runx3 specifically in MNP were generated by crossing *Runx3*^*fl/fl*^ mice [18] onto *CD11c-Cre* [20] or *Cx3cr1c-Cre* mice [21], giving rise to *Runx3*^*fl/fl*^*/CD11c*:*Cre* (named *Runx3*^**Δ**^) or *Runx3*^*fl/fl*^*/Cx3cr1*:*Cre* (*Cx3cr1-Runx3*^*Δ*^) mice, respectively. *Runx3*^**Δ**^ mice were crossed onto *Cx3cr1-GFP* mice [22] to obtain *Runx3*^**Δ**^ Cx3cr1-GFP mice. All animals were on the C57Bl/6 background.

Mice lacking Runx3 specifically in T cells were generated by crossing *Runx3*^*fl/fl*^ mice onto *Lck-Cre* mice [23] (*Runx3*^*fl/fl*^*/Lck*:*Cre)*. The *Runx3*^*P1-AFP/P2-GFP*^ knock-in mice referred to as Runx3-GFP and *Runx3*^*-/-*^ mice in the present study have been previously described [18]. C57Bl/6 Ly5.2 mice were purchased from Harlan Laboratories (Israel). C57Bl/6 Ly5.1 mice were bred in the Weizmann Animal Facility. To determine the ability of Runx3^-/-^ leukocytes to transfer colitis, we adoptively transferred intravenously 3x10^6^ E13.5 fetal liver (FL) cells from WT or *Runx3*^*-/-*^ C57Bl/6 mice into lethally irradiated (800R and 400R, 4 h apart) C57Bl/6 mice and colitis pathology was determined 2 months after transfer. For generation of BM chimeras, C57Bl/6 CD45.1 mice were lethally irradiated (1050R) and reconstituted by intravenous injection of a 1:1 mixture of WT C57Bl/6 CD45.1 and CD11c-Runx3^Δ^ CD45.2 BM cells. Mice were analyzed 11–15 weeks post BM transfer. Animals were maintained under specific pathogen free (SPF) conditions and handled in accordance with the protocol approved by the Institutional Animal Care and Use Committee (IACUC) of the Weizmann Institute of Science (Permit #: 09750119–4). Specifically, mice were kept at 22±1°C and 50±10% humidity in individually ventilated cages at the Weizmann Instititute animal facility. Diet of ENVIGO Teklad rodent diet, Hydropac water (2.6–2.7 PH level) and environmental enrichment with Nestlets and cardboard houses. Mice were sacrificed by CO_2_ euthanasia and cervical dislocation. Routine monitoring was conducted by trained animal care personnel during the entire hosting period of the mice. Sentinels are routinely used to monitor the mouse colonies for the presence of pathogens and are examined after two months. A veterinarian is informed of any unexpected health issue of mice.

Genotyping primers; Floxed: F5’-CCCACCCATCCAACAGTTCC, R5’-GAGACCACAGAGGACTTGTA. CRE: F5’-AACATGCTTCATCGTCGG, R5’-TTCGGATCATCAGCTACACC. Cx3cr1-GFP mice WT or GFP allele: F5’-TTCACGTTCGGTCTGGTGGG, R5’- GGTTCCTAGTGGAGCTAGGG and F5’-GATCACTCTCGGCATGGACG, R5’-GGTTCCTAGTGGAGCTAGGG, respectively. Runx3-P1 AFP: F5’-TCTCGCGTTCTTTCCCCATTTTT. R3’-GCCGGTGGTGCAGATGAACT. Runx3-P2 GFP: F5’-AGCCGCCGCCTTCCCGCC, R3’-TGGTGCAGATGAACTTCAGG.

### Isolation and analysis of colonic LP cells

Isolation of colonic LP cells was performed as previously described with some modifications [24]. Briefly, extra-intestinal fat tissue and blood vessels were carefully removed and colons were then flushed of their luminal content with cold PBS. Cecum was sectioned longitudinally, and cut into 0.5 cm pieces. Epithelial cells and mucus were removed via 40 min incubation at 37°C with shaking at 250 rpm in Hank’s balanced salt solution containing 5% fetal bovine serum (FBS) and 2 mM EDTA. Colon pieces were then digested by 40 min incubation at 37°C with shaking in RPMI-1640 containing 5% FBS, 1 mg/ml Collagenase II (Worthington, US) and 0.1 mg/ml DNase I (Roche, US). The digested cell suspension was then washed with PBS and passed through a 100μm cell strainer. For analysis of blood monocytes, samples were resuspended in ACK erythrocyte-lysis buffer (0.15M NH4Cl, 0.1M KHCO3 and 1mM EDTA in PBS). For intracellular staining, a Foxp3 buffer set (Invitrogen, US) was used according to manufacturer instructions. To induce T-cell activation for determination of intracellular IFN- γ, LP cells were incubated for 5 h with 20 ng/ml TPA and 1 μg/ml ionomycin in the presence of 10 μg/ml Brefeldin A (Sigma, IL). For fluorescence-activated cell sorting (FACS) analysis, single cell suspensions were stained with the following specified antibodies; Epcam G8.8, CD16/32 clone 93, CD45 30-F11, MHCII M5/114.15.2, CD11c N418, Ly6c HK1.4, CD11b M1/70, CD103 2E7, F4/80 BM8, CD64 X54-5/7.1, CD115 AFS98, CD43 eBioR2/60, Foxp3 MF-14, CD25 PC61, CD45RB C363-16A, CD4 RM4-5, Clec12a 5D3, PD-L2 TY25, CD24a M1/69, T-bet 4B10, IFN-γ XMG1.2, CD45.1 A20 and CD45.2 104. All Abs were purchased from BioLegend, US or eBiosciences, US unless indicated otherwise. LSRII flow cytometer (BD Biosciences, US) with FACSDiva Version 6.2 software (BD Biosciences, US) was used and further data analysis was conducted using FlowJo software. FACSAria flow cytometer (BD Biosciences, US) was used for cell sorting and forward scatter height versus forward scatter width appearance was used to exclude doublets.

### Immunofluorescence (IF) and Immunohistochemistry (IHC)

Colon was removed and snap-frozen in liquid nitrogen. Cryo-sections of 12–14 μm were prepared on glass slides, fixed for 3 min in acetone at -20°C and air dried for 20 min. Slides were blocked with PBS containing 20% horse serum and stained overnight at room temperature with rabbit anti-MHCII-biotin Ab followed by incubation with SA-cy3 Ab. MHCII staining and endogenous Cx3cr1-GFP signal were analyzed under a fluorescent microscope. In addition, 4μm serial paraffin sections of colon and stomach were prepared and stained with H&E for histopathology evaluation as described [17].

### RNA extraction and microarray gene expression analysis

Total RNA was extracted from sorted cecal MNP using the RNeasy Micro Kit (QIAGEN, US). In each experiment sorted cells were pooled from 3–4 mice. BioAnalyzer 2100 (Agilent Technologies, US) was used to determine RNA quality. RNA from each sample was labeled and hybridized to Affymetrix mouse exon ST 1.0 microarrays according to manufacturer instructions. Microarrays were scanned using GeneChip scanner 3000 7G. Statistical analysis was performed using the Partek^®^ Genomics Suite software (Partek Inc., US). CEL files (raw expression measurements) were imported to Partek GS and data was preprocessed and normalized using the RMA algorithm [25] with GC correction. To identify DEGs, a one-way ANOVA test was applied. DEGs lists were created by filtering the genes based on an absolute fold change ≥ 1.5, p ≤ 0.05. Log_2_ gene intensities were used for volcano scatter plots (Partek). Ingenuity software was used for GO analysis. The GEO accession number is presented in the next section.

### Chromatin Immunoprecipitation Sequencing (ChIP-seq) data acquisition and analysis

Two biological replicate ChIP-seq experiments were conducted for detection of Runx3-bound genomic regions. 30x10^6^ cells of the D1 DC cell line were fixed in 1% formaldehyde and sonicated to yield DNA fragments of ~300 bp according to standard procedures previously described [26]. For immunoprecipitation (IP), 40 μl of in-house anti-Runx3 Ab were added to 15 ml of diluted fragmented chromatin and incubated overnight at 4°C; Rabbit pre-immune serum was used as control. DNA was purified using QIAquick spin columns (QIAGEN, US). For ChIP-seq analysis, Illumina sequencing of short reads was performed using an Illumina Genome Analyzer. For data analysis, extracted Runx3 IP and control IP sequences were aligned uniquely to the mouse genome (mm9) using the bowtie software [27]. Bound regions were detected using the Model-based Analysis of ChIP-Seq (MACS2) [28]. Runx3 bound peaks and coverage data (bigWig files) were uploaded to the UCSC genome browser. The GREAT algorithm (version 3.0.0) [29] was applied to determine genes corresponding to Runx3-bound peaks in D1 cells and splenic CD4^+^ DCs (GSE48588) [14] as well as those corresponding to H3K4me1, H3K27Ac and ATAC-seq peaks in colonic RM (GSE63340) [30]. Cistrome CEAS platform [31] was used to compute enrichment of genomic sequences. Discovery of TF binding sites was conducted using Genomatix analysis software (https://www.genomatix.de). All microarray and ChIP-seq data are available in the GEO public database under the SuperSeries accession number GSE136067.

### RT–qPCR and protein analysis

Total RNA was reverse-transcribed using Omniscript reverse transcription kit (QIAGEN, US) according to manufacturer’s instructions. Quantitation of cDNAs was performed by applying sequence-specific primers, miScript SYBR Green PCR kit (QIAGEN, US) and using Roche LC480 LightCycler. Target transcript quantification was calculated relative to *Hprt* mRNA. Standard errors were calculated using the Relative Expression Software Tool (REST) [32].

qPCR Primers:

Runx3 ex3-4 F: 5’- GCCGGCAATGATGAGAACT
Runx3 ex3-4 R: 5’- CACTTGGGTAGGGTTGGT
Hprt-F: 5’- GTTGGATACAGGCCAGACTTTGTTG
Hprt-R: 5’- CCAGTTTCACTAATGACACAAACG
Hpgds-F: 5’- GGAAGAGCCGAAATTATTCGCT
Hpgds-R: 5’- ACCACTGCATCAGCTTGACAT
Ccl24-F: 5’- ACCGAGTGGTTAGCTACCAGTTG
Ccl24-R: 5’- TGGTGATGAAGATGACCCCTG
Fcrls-F: 5’- ACAGGATCTAAGTGGCTGAATGT
Fcrls-R: 5’- CTGGGTCGTTGCCCTATCTG
Cxcl9-F: 5’- TCCTTTTGGGCATCATCTTCC
Cxcl9-R: 5’- TTTGTAGTGGATCGTGCCTCG
Ms4a14-F: 5’- ACCAACAGACCAGCAGTCAGAAGA
Ms4a14-R: 5’- TTGGATGAGCCTGAGCAAGGTGTA
Pd-l2 –F: 5’- CTGCCGATACTGAACCTGAGC
Pd-l2 –R: 5’- GCGGTCAAAATCGCACTCC

### Protein analysis

Cell proteins were extracted with Radioimmunoprecipitation assay (RIPA) buffer containing protease inhibitors and analyzed by western blotting using either in-house anti-Runx3 or anti-Runx1 Ab. Emerin was used as an internal loading control.

### Statistical analysis

Statistical significance of FACS data was determined using the unpaired, two tailed Student’s t-test. Statistical significance of enrichment of ChIP-seq peaks associated with Runx3^Δ^ RM DEGs versus non-DEGs was analyzed on expressed genes (Log2 ≥ 5 in at least one of seven samples) using contingency tables and calculating significance by the Fisher exact test and Pearson’s Chi-squared test with Yates’ continuity correction by the R package.

## Results

### Loss of Runx3 in non-T cells leukocytes induces colitis

**Fig 1:**
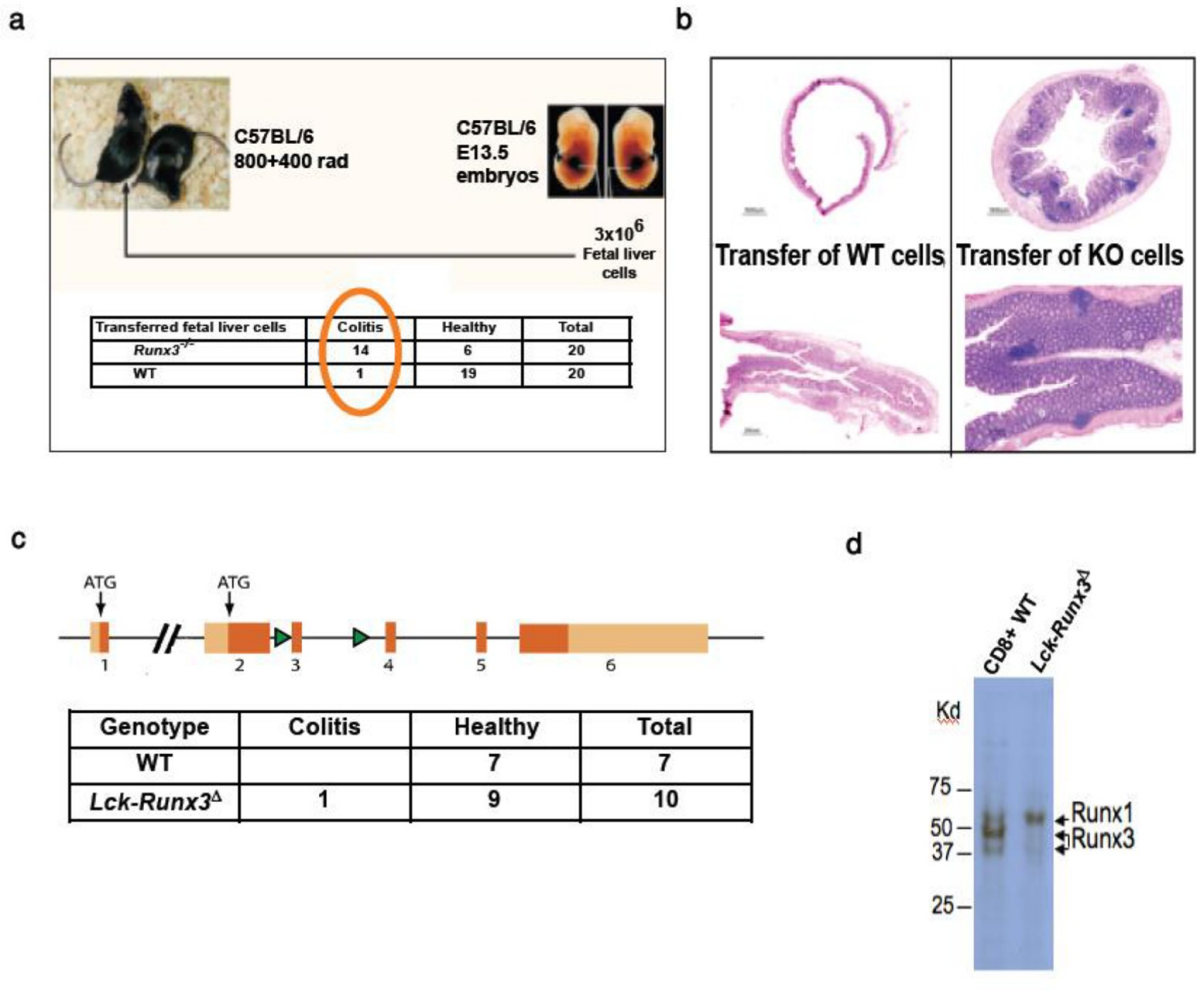
Loss of Runx3 in T cells does not induce colitis. (a) Number of recipient mice with colitis. Colitis was scored on a scale of 1 to 4 (1, minimal; 2, mild; 3, moderate; and 4, severe). Recipients of *Runx3*^*-/-*^ FL cells developed on average mild colitis (b) Histological sections of colon stained with hematoxylin and eosin (H&E). (c) Upper, schematic representation of the *Runx3* gene locus targeted for conditional inactivation. Dark orange boxes represent coding exons; light orange boxes represent untranslated regions (UTRs); exons are marked by numbers. Green arrowheads represent lox-P sites that flank exon 3, one of the exons comprising the RUNX TF DNA binding domain. Lower, T cell-specific ablation of Runx3 (*Lck-Runx3*^**Δ**^) does not induce colitis, as shown in the table. (d) Western blot of CD8^+^ T cell extract from WT and *Lck-Runx3*^**Δ**^ mice. Note the efficient deletion of Runx3 and up-regulation of Runx1 in *Lck-Runx3*^**Δ**^ cells.

To establish whether Runx3^-/-^ leukocytes are involved in colitis development, we performed a transfer experiment. Because *Runx3*^*-/-*^ mice on a C57Bl/6 background are not viable after birth, we adoptively transferred E13.5 fetal liver (FL) cells from either *Runx3*^*-/-*^ or WT mice into lethally irradiated C57Bl/6 mice. Histopathological analysis performed 2 months after transfer revealed that recipients of Runx3^-/-^, but not of WT FL cells, developed inflammatory bowel disease (IBD) (Fig 1a and 1b). These findings are consistent with those of Sugai et al [33].

To determine whether this phenotype could be attributed to T cells, we crossed *Runx3*^*fl/fl*^ mice on to *Lck*-*Cre* mice, to obtain T cell-specific Runx3 conditional knockout (cKO) mice. Unlike the spontaneous colitis seen in *Runx3*^*-/-*^ mice, *Lck-Runx3*^***Δ***^ mice did not develop spontaneous colitis (Fig 1c and 1d). As indicated earlier, colitis was not evident in *NKp46-Runx3*^***Δ***^ mice either [12]. These results indicate that Runx3 function in T, NK cells and ILC is not essential to maintain GIT homeostasis, and suggest that cell-autonomous Runx3 function in other immune cell types, possibly MNP, is involved.

### Mice lacking Runx3 in colonic MNP develop spontaneous early-onset colitis

The MNP are known for their role in maintaining GIT homeostasis [34]. In order to determine whether Runx3 expression in MNP plays a role in GIT immune tolerance, we generated MNP-specific Runx3-cKO mice by crossing *Runx3*^*fl/fl*^ mice with two different Cre transgenic strains, *CD11c-Cre* and *Cx3cr1-Cre*, generating *Runx3*^**Δ**^ and *Cx3cr1-Runx3*^**Δ**^ mice, respectively. Both CD11c and Cx3cr1 are expressed in DC and macrophages, but CD11c is expressed at a higher level in DC and Cx3cr1 is expressed at a higher level in macrophages.

**Fig 2:**
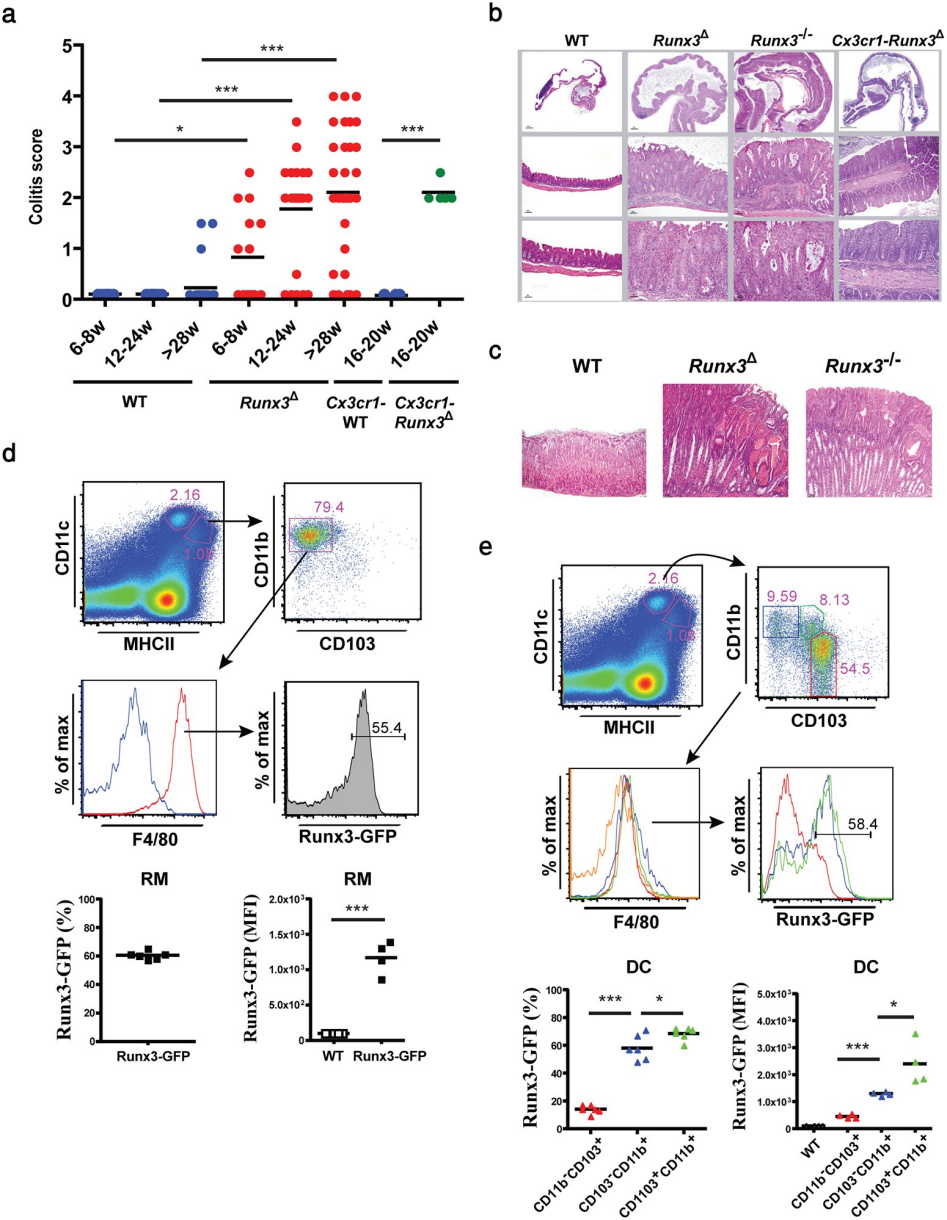
Colitis development in *Runx3*^*-/-*^ and in the two MNP-specific *Runx3*-cKO mice (*Runx3*^Δ^ and *Cx3cr1-Runx3*^Δ^). (a) A graphical summary of postmortem colitis grade in *Runx3*^**Δ**^ and *Cx3cr1-Runx3*^**Δ**^ (right) relative to WT mice (left). Colitis was scored on a scale of 1 to 4 (1, minimal; 2, mild; 3, moderate; and 4, severe). Each dot represents a single mouse. (note the complete absence of pathological signs of colitis in 24 weeks-old WT mice). (b) H&E stained histological sections of colons from WT and the three Runx3-deficient mouse strains. (c) H&E stained histological sections of stomach of two one-year old Runx3-deficient mouse strains compared to WT control. (d) Flow cytometry analysis of *Runx3* expression in colonic RM of Runx3-GFP^+^ mice. RM were identified by F4/80 expression (red line) and overlay on F4/80^-^CD11b^+^ (blue line) (top and middle panels). Graphical summary of Runx3-GFP^+^ RM (bottom, left panel) and mean fluorescence intensity (MFI) relative to non-GFP (WT) control (bottom, right panel). (e) Flow cytometry analysis of *Runx3* expression in colonic CD103^+^CD11b^-^ cDC1 (red), CD103^-^CD11b^+^ cDC2 (blue) and CD103^+^CD11b^+^ cDC2 (green) (top and middle panels). Orange line represents the F4/80 negative level. Graphical summary of Runx3-GFP^+^ prevalence among the DC subsets (bottom left) and Runx3-GFP^+^ MFI relative to non-GFP (WT) control (bottom right). Dot plots horizontal bars represent mean values, two-tailed Wilcoxon test (Fig 2a) and unpaired two-tailed t-test (Figs 2d and 2e). * p<0.05, *** p<0.001.

Strikingly, the majority of ~3-month-old mice of both MNP-specific *Runx3*-cKO models spontaneously developed mild colitis that affected the cecum and proximal colon (Fig 2a and 2b).

Colitis in these *Runx3*-cKO mice displayed similar characteristics to those observed at an earlier age in *Runx3*^*-/-*^ mice [17], including accumulation of infiltrating leukocytes associated with pronounced mucosal hyperplasia and loss of differentiated mucous secreting goblet cells. *Runx3*^**Δ**^ and *Cx3cr1-Runx3*^**Δ**^ mice older than 7 months typically showed exacerbated colon inflammation and 30% of them also developed gastropathy (Fig 2c), resembling the phenotype of *Runx3*^*-/-*^ mice [17]. Of note, younger mice (6–8 weeks of age) showed low colitis prevalence with a minimal average disease score. As colitis developed in the MNP-specific *Runx3*-cKO mice, we determined which subtype of colonic lamina propria (LP) MNP cells expresses *Runx3* by employing Runx3-GFP compound heterozygous mice described previously [14]. Our analysis revealed that within the LP MNP compartment, *Runx3* is expressed in RM, characterized as CD11c^int^CD11b^+^MHCII^+^F4/80^+^CD103^-^ (Fig 2d), and also at a low level in LP CD11c^lo^ monocytes (S1a Fig), but not in circulating monocytes (CD11b^+^CD115^+^CD43^hi^CD11c^+^Ly6c^-^ and CD11b^+^CD115^+^CD43^lo^CD11c^-^Ly6c^+^) (S1b Fig). These results correspond with the identification of Runx3 as an intestinal RM-specific TF [30] and indicate that Runx3 expression accompanies the differentiation of monocytes to colonic RM.

In addition to RM, the MNP compartment contains three DC subsets, commonly characterized as CD11c^high^MHCII^+^F4/80^low^. These subsets include CD103^+^CD11b^-^ termed conventional DC1 (cDC1) and two cDC2 subsets: CD103^-^CD11b^+^ and CD103^+^CD11b^+^ DC. We detected expression of *Runx3* in the majority of CD103^-^CD11b^+^ and CD103^+^CD11b^+^ DC, while only a small fraction of cDC1s expressed *Runx3*. Moreover, these *Runx3*-expressing cDC1s had a lower *Runx3* level relative to the two CD11b^+^ DC subsets (Fig 2e).

Recent studies on the relationship between intestinal DC subsets suggested that CD103^+^CD11b^+^ DC develop from intermediate CD103^-^CD11b^+^ DC in a TGFβ-dependent manner [35]. Accordingly, CD103^+^CD11b^+^ DC expressed higher *Runx3* levels than CD103^-^CD11b^+^ DC (Fig 2e). Taken together, our results suggest that Runx3^-/-^ CD11b^+^ MNPs, including RM and CD11b^+^ DC, drive the spontaneous development of colitis in *Runx3*^*-/-*^ mice. Nevertheless, the possible contribution of the small fraction of Runx3-expressing CD103^+^CD11b^-^ DC cannot be ruled out. Using *Runx3-P1*^*AFP/+*^ or *Runx3-P2*^*GFP/+*^ reporter mice we established that while both promoters mediate *Runx3* expression in CD103^+^CD11b^+^ DC and in RM, P1 or P2 were preferentially used in these cells, respectively (S1c Fig). Collectively, these results indicate that the known role of intestinal MNP in maintenance of GIT homeostasis [34] is mediated, at least in part, by P1- and P2-driven *Runx3* expression.

### Loss of Runx3 in MNP causes an early imbalance of colonic MNP subsets

As loss of Runx3 in MNP triggers spontaneous colitis, we sought to determine whether alterations in the colonic MNP compartment of *Runx3*^**Δ**^ mice precede the onset of colitis. Colonic RM are derived from recruited circulating monocytes, which differentiate to RM in a four stage (P1 to P4) “waterfall” process [36, 37].

**Fig 3:**
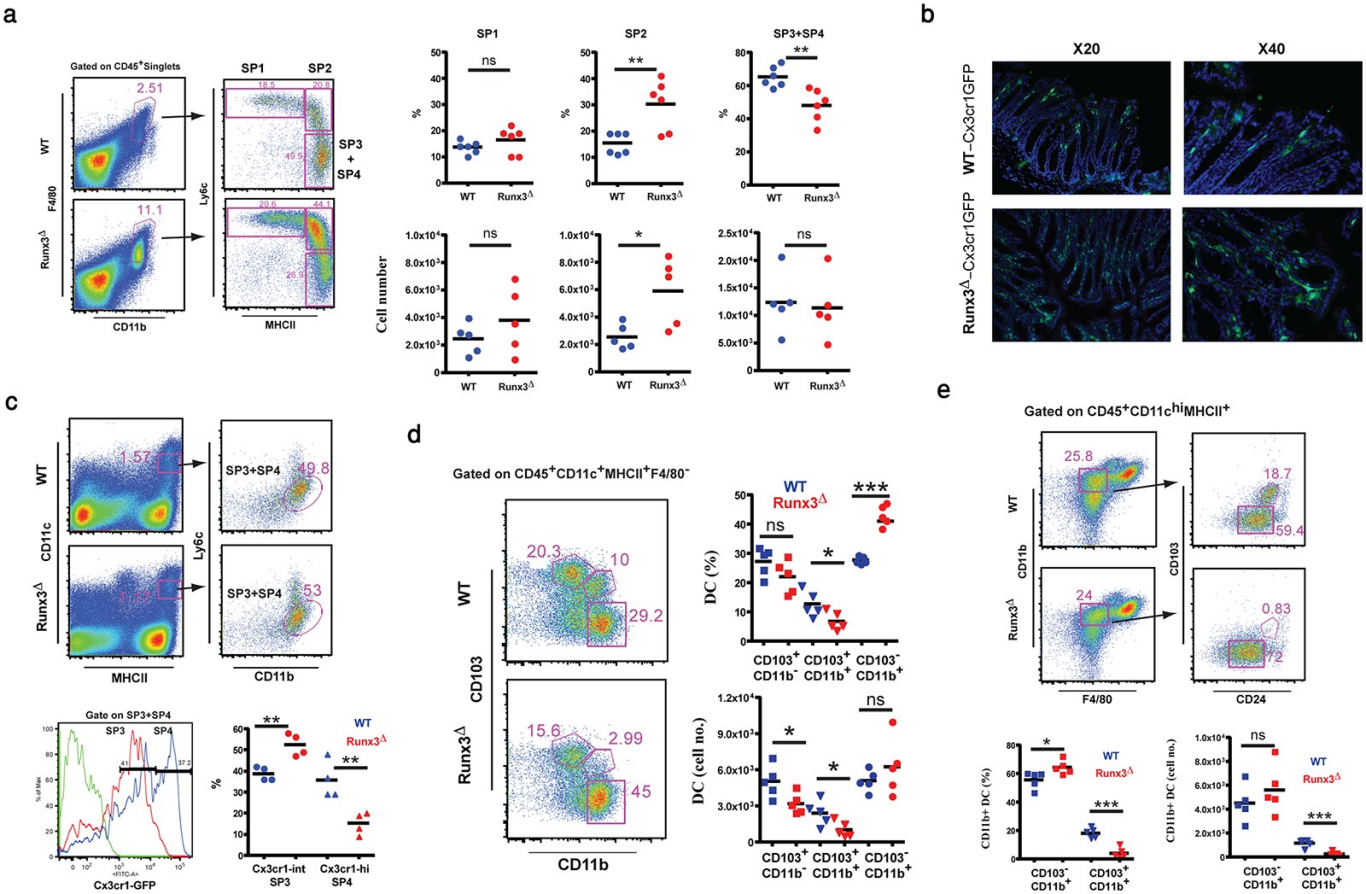
Loss of Runx3 in MNP causes an imbalance among colonic MNP subsets. (a) Comparison of WT and *Runx3*^**Δ**^ colonic monocyte-macrophage “waterfall”. Representative flow cytometry gating on CD45^+^CD11b^+^F4/80^+^ cells demonstrating P1, P2 and P3+P4 cells (left). Graphical summary comparing the prevalence and cell numbers of WT and *Runx3*^***Δ***^ colonic P1, P2 and P3+P4 waterfall cells (right). (b) GFP signal in frozen section of the colon of 6-8-week-old *Runx3*^**Δ**^-*Cx3cr1*^*GFP/+*^ mice compared to WT-Cx3cr1^GFP/+^ littermates. (c) Analysis of Cx3cr1-GFP expression level in WT RM relative to *Runx3*^**Δ**^ colonic RM. Top, representative flow cytometry gating on WT and *Runx3*^**Δ**^ CD45^+^CD11c^int^MHCII^+^CD11b^+^Ly6c^-^ RM. Bottom, overlaid histogram (left) of Cx3cr1-GFP expression level in WT (blue) and *Runx3*^**Δ**^ (red) RM and graphical summary (right) of the frequency of Cx3cr1^Hi^ and Cx3cr1^Int^ macrophages in WT and Runx3^**Δ**^ mice. (d) DC subsets in WT and *Runx3*^**Δ**^ colonic LP of 7-8-week-old mice. Left, a representative flow cytometry of the CD103^+^CD11b^-^, CD103^+^CD11b^+^ and CD103^-^CD11b^+^ DC subsets. Right, graphical summary depicting the prevalence and cell numbers of the three colonic LP DC subsets. (e) Representative flow cytometry gating on CD45^+^CD11c^hi^MHCII^+^CD11b^+^F4/80^lo^ cells comparing CD24a expression between *Runx3*^**Δ**^ and WT at 8 weeks of age (upper). Graphical summary comparing the prevalence and cell numbers of WT and Runx3^**Δ**^ CD103^+^CD11b^+^CD24a^+^ and CD103^-^CD11b^+^CD24a^+^ DC (lower). Dot plots horizontal bars represent mean values, unpaired two-tailed t-test * p<0.05, **p<0.01, ***p<0.001.

Under steady-state conditions in WT mice, the mature anti-inflammatory P3 and P4 stages predominated (Fig 3a). In *Runx3*^**Δ**^ mice, at 6–8 weeks of age there was a marked increase in the prevalence of Ly6c^+^ pro-inflammatory P2 monocytes with a concomitant decrease in the mature P3+P4 fraction (Fig 3a). These results suggest that Runx3 function in MNP is required for the maturation of anti-inflammatory RM and loss of Runx3 in RM leads to increase in LP monocytes.

The transition from P3 to fully mature P4 RM is characterized by increased expression of Cx3cr1 [37]. To comparatively analyze Cx3cr1 expression levels in colonic Runx3^**Δ**^ *versus* WT RM, we generated *CD11c-Runx3*^**Δ**^*-Cx3cr1*^*GFP*^ mice. Examination of the GFP signal in *Runx3*^**Δ**^ and WT colonic sections revealed higher GFP intensity in the colon of the *Runx3*^**Δ**^, indicating that *Runx3*^**Δ**^ LP harbor elevated numbers of Cx3cr1-expressing cells. Furthermore, while GFP-positive cells in WT colon sections tended to juxtapose the epithelium, the GFP signal in the *Runx3*^**Δ**^ colon sections was evenly scattered in the LP, (Fig 3b). This difference in localization of LP Cx3cr1-expressing cells between WT and *Runx3*^**Δ**^ mice reflects the difference in the inflammatory condition. Under non-inflammatory condition the WT RM are positioned in close contact with the epithelium and their Cx3cr1 expression enables production of the trans-epithelial dendrites and access to the lumen. The more scattered appearance of the Cx3cr1^+^ cells in *Runx3*^**Δ**^ mice represents the increase in recruited monocytes that differentiate to macrophages in the LP, which characterizes the inflammatory state. Still and more, GFP intensity analysis revealed a significant decrease in the number of Cx3cr1^GFP-hi^ P4 cells and an increased number of the less mature Cx3cr1^GFP-int^ P3 cells in the *Runx3*^**Δ**^ LP (Fig 3c and S2a Fig). Taken together, these results indicate that Runx3 is involved in the terminal maturation of RM from the P3 to the anti-inflammatory P4 stage.

Analysis of 7–8 weeks-old *Runx3*^**Δ**^ mice revealed that among the three DC subsets, the CD103^+^CD11b^+^ subset was decreased and the CD103^-^CD11b^+^ DC subsets was increased, compared with their WT littermates, while the CD103^+^CD11b^-^ DC subset was only slightly reduced. Furthermore, quantification of DC subsets displayed a significant reduction in the number of CD103^+^CD11b^+^ DC in the *Runx3*^**Δ**^ mice (Fig 3d). Employing CD24a, another marker of CD103^+^CD11b^+^ DC, detected an even more substantial reduction in prevalence and number of CD103^+^CD11b^+^ DC in *Runx3*^**Δ**^ mice (Fig 3e). To determine whether the above documented reduction in the abundance of CD103^+^CD11b^+^ DC in *Runx3*^**Δ**^ compared to WT mice occurs earlier than at 7–8 weeks, we examined 5–6 weeks-old mice. Importantly, at this younger age the *Runx3*^**Δ**^ mice showed no difference in DC subsets distribution and cell numbers compared to WT mice (S2b Fig). These findings correspond with the pathology data in Fig 2a. In addition, *Runx3*^**Δ**^ CD103^+^CD11b^+^ DC displayed reduced levels of both CD24a and CD103 (S2b and S2c Fig). Thus, these results suggest that loss of Runx3 in *Runx3*^**Δ**^ mice causes an imbalance among colonic MNP subsets prior to the onset of significant colitis. These changes are associated with reduction in the anti-inflammatory mature RM and DC2 populations, reflected by the increased prevalence of colonic P2 monocytes and CD103^-^CD11b^+^ DC and the decrease in anti-inflammatory P4 RM and CD103^+^CD11b^+^ DC.

Overall, the findings support a cell-autonomous Runx3 function in colonic CD11b^+^ MNP, emphasizing their importance to intestinal homeostasis. Accordingly, loss of Runx3 in MNP causes an early impairment of colonic CD11b^+^ MNP, marking it a sign of early-onset GIT inflammation.

### Transfer of WT BM overcomes the onset of Runx3^Δ^ BM-mediated colitis

Competitive BM-repopulation assays were conducted to address whether Runx3-sufficient MNP regulate an anti-inflammatory response or whether in the presence of WT MNP, Runx3^**Δ**^ MNP will still dictate a pro-inflammatory response. Reconstituted mice colons were examined histopathologically for signs of colitis, and by flow cytometry to assess the replenishment of colonic MNP compartment. A 1:1 mixture of Runx3^**Δ**^ BM CD45.2 and WT BM CD45.1 cells was transferred into WT CD45.1 recipient mice.

**Fig 4:**
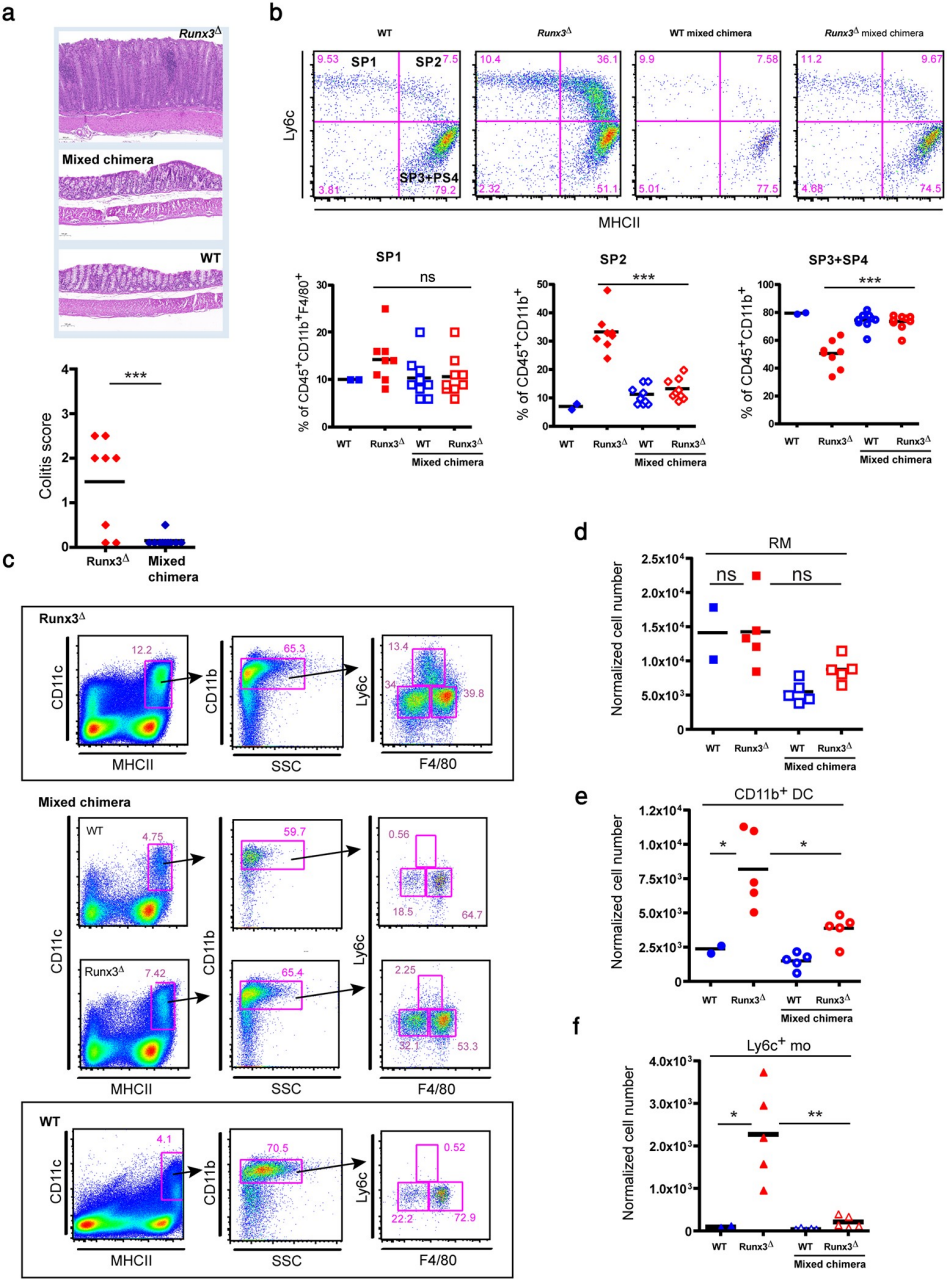
Colitis and colonic MNP subsets in lethally irradiated mice reconstituted with WT, *Runx3*^Δ^ or a mixture of WT/*Runx3*^Δ^ BM cells. (a) Representative H&E stained colon section of Runx3^**Δ**^ BM recipient mice [Runx3^**Δ**^ (CD45.2) →WT(CD45.1)], mixed chimera [Runx3^**Δ**^ (CD45.2)/WT (CD45.1)→ WT(CD45.1)] and WT BM recipient mice [WT (CD45.1)→WT (CD45.1)] (top). Colitis score was scaled from one to four (1-minimal; 2-mild; 3-moderate; 4-severe), (bottom). (b) Comparison of colonic LP P1 to P4 waterfall between WT, Runx3^**Δ**^ and mixed chimeric mice (WT and Runx3^**Δ**^) BM recipient mice. Representative waterfall from each group (top) and summary of P1, P2 and P3+P4 abundance in the three groups (bottom) are shown. (c) Representative flow cytometry profile of colonic LP MNP subsets in *Runx3*^***Δ***^ BM recipient mice (top), mixed chimeric mice (middle) and WT BM recipient mice (bottom). (d) Graphical summary of colonic LP RM normalized cell number in reconstituted BM mixed chimeric mice compared to Runx3^**Δ**^ BM reconstituted mice and WT BM reconstituted mice. (e) Graphical summary of colonic LP CD11b^+^ DC normalized cell number in reconstituted BM mixed chimeric mice compared to Runx3^**Δ**^ BM reconstituted mice and WT BM reconstituted mice. (f) Graphical summary of colonic LP Ly6c^+^ monocytes normalized cell number in reconstituted BM mixed chimeric mice compared to Runx3^**Δ**^ BM recipient mice and WT BM recipient mice. Normalization was to 10^5^ CD45^+^ cells. Dot plots horizontal bars represent mean values, unpaired two-tailed t-test * p<0.05, ** p<0.01, *** p<0.001.

Eleven to fifteen weeks following transfer, colons of experimental mixed chimeric (Runx3^**Δ**^/WT→WT) mice were compared to those of Runx3^**Δ**^→WT and WT→WT reconstituted mice. No differences in weight gain were observed between the different recipient mice groups during the course of the transfer experiment (S3a Fig). Colons of Runx3^**Δ**^ BM reconstituted mice displayed colitis development (Fig 4a). Interestingly, however, the mixed chimeric BM reconstituted mice did not develop colitis (Fig 4a). This finding underscores the ability of Runx3-sufficient MNP to dictate a mucosal anti-inflammatory environment that overrides the pro-inflammatory state of Runx3^**Δ**^ MNP.

Analysis of colonic RM waterfall in Runx3^**Δ**^ BM recipients revealed a marked increase in the P2 fraction and a concomitant decrease in the P3+P4 macrophage fraction. These finding are consistent with our observations in *Runx3*^**Δ**^ mice and reports on other mouse strains and IBD development [37, 38]. In accordance with their normal phenotype, the BM mixed chimera replenished mice exhibited a normal RM waterfall resembling that of the WT BM transferred mice (Fig 4b). Yet, despite the normal colon histology in the mixed chimeric recipient mice, flow cytometry analysis comparison revealed differences in abundance of WT and Runx3^**Δ**^ LP MNP in mixed chimeric mice. While mixed chimera WT/CD45.1 MNP consisted of 75% RM and ~20% CD11b^+^ DC, mixed chimera Runx3^**Δ**^/CD45.2 MNP displayed a reduction in number of RM to 60%, and a concomitant increased proportion of CD11b^+^ DC to 35% (Fig 4c and S3b Fig). Notably, RM and CD11b^+^ DC subsets distribution in chimeric mice (WT/CD45.1 and *Runx3*^**Δ**^/CD45.2) were similar to those of corresponding subsets in WT and Runx3^**Δ**^ BM replenished mice, respectively. However, mixed chimeric Runx3^**Δ**^ MNP mice displayed a reduction in number of Ly6c^+^ colonic monocytes, compared to mice reconstituted only with *Runx3*^**Δ**^ BM (S3b Fig).

We then analyzed colonic normalized cell numbers for each subpopulation. Mice reconstituted with Runx3^**Δ**^ BM had a similar RM cell number as mice reconstituted with WT BM (Fig 4d). In contrast, Runx3^**Δ**^ BM reconstituted mice had a substantially increased number of CD11b^+^ DC and Ly6c^+^ monocytes (Fig 4e and 4f). Importantly, mixed chimeric *Runx3*^**Δ**^/CD45.2 mice displayed a significant reduction in number of CD11b^+^ DC and Ly6c^+^ monocytes, compared with Runx3^**Δ**^ BM reconstituted mice (Fig 4e and 4f).

These results reveal that Runx3-sufficient MNP suppressed the influx and propagation of Runx3^**Δ**^ monocytes and CD11b^+^ DC by imposing an anti-inflammatory environment, which prevented the induction of colitis by the pro-inflammatory Runx3^Δ^ MNP. These phenomena were reflected by the re-established equilibrium within the MNP compartment of mixed chimeric mice.

### *Runx3*^Δ^ colonic RM transcriptome displays an anti-inflammatory to pro-inflammatory switch and colonic MNP maturation defect

The observations presented above revealed that while colonic RM and CD11b^+^ DC are formed in the absence of Runx3, *Runx3*^**Δ**^ mice can still develop colitis. To gain further insight into the Runx3-dependent transcriptional program controlling intestinal MNP homeostatic function, we analyzed the transcriptomes of both WT and Runx3^**Δ**^ RM and CD11b^+^ DCs. RNA of 6-8-week-old mice was prepared from sorted cecum RM and CD11b^+^ DC (S4a Fig).

**Fig 5:**
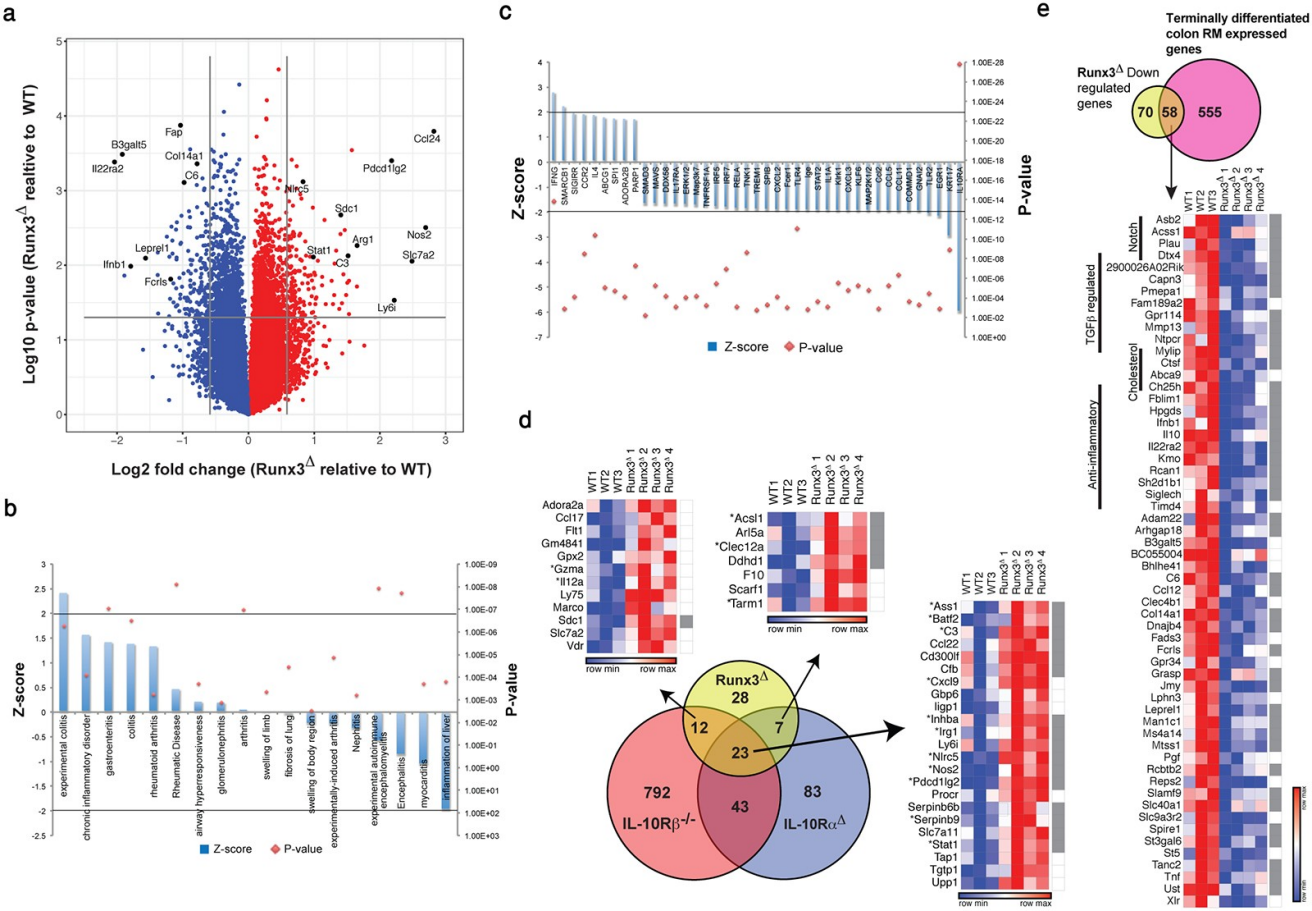
Runx3^Δ^ RM transcriptome reveals impaired maturation and up-regulation of pro-inflammatory genes as occurs in Il10r^Δ^ RM. (a) A volcano plot of colon RM DEGs in 6–8 weeks old *Runx3*^**Δ**^ and WT control mice. Numerous up- or down-regulated genes in Runx3^**Δ**^ are indicated. (b) GO analysis categorized by "disease and disorder". (c) GO analysis categorized by "upstream regulators". (d) Venn diagram (lower part) representing the overlap between Runx3^**Δ**^, Cx3cr1-Il10ra^***Δ***^ and Il10rb^*-/-*^ RM up-regulated genes. Cutoff for Runx3^**Δ**^ and the two Il10r-deficient RM DEGs was set to 1.5 and 2-fold change, respectively. Heat maps representing the standardized expression values of common genes; asterisks mark the pro-inflammatory genes. Runx3 high-confidence targets are represented by gray squares in the right column. (e) Venn diagram representing the overlap between down-regulated genes in *Runx3*^**Δ**^ RM and up- regulated genes in terminally differentiated P4 RM vs. their P1 monocyte precursors (top). Standardized expression values of shared genes are specified in the heat map (bottom). Runx3 high-confidence targets are represented by right column gray squares.

Principal component analysis (PCA) of transcriptome data including all the indicated samples, revealed two distinct cell populations corresponding to CD11b^+^ DC and RM (S4b Fig). Analysis of differential gene expression between Runx3^**Δ**^ and WT RM revealed 72 up-regulated and 128 down-regulated genes in Runx3^**Δ**^ (fold-change ≥ 1.5, p-value ≤ 0.05) (Fig 5a and S1 Table sheet1).

Several of these Runx3^**Δ**^ RM differentially expressed genes (DEGs) were validated by RT-qPCR (S4c Fig). In addition, flow cytometry analysis confirmed the up-regulation of two surface proteins encoded by Runx3^**Δ**^ RM DEGs *Pdcd1lg2* and *Clec12a* (S4d and S4e Fig). Interestingly, gene ontology (GO) enrichment analysis of these DEGs using the term “disease and disorder” yielded “experimental colitis” as the top enriched term in "inflammatory/auto-immune disease" category (Fig 5b). In line with the GO enrichment for the term "colitis", expression of inflammatory genes was affected; 19 out of 21 pro-inflammatory DEGs (85%) were up-regulated and 14 out of 19 (74%) anti- inflammatory DEGs were down-regulated, respectively, in Runx3^**Δ**^ RM (Table 1). These results indicate that loss of Runx3 in MNP switches colonic RM from an anti-inflammatory to a pro-inflammatory state. GO analysis to detect potential upstream regulators of Runx3^**Δ**^ RM DEGs highlighted IL10RA and IFNG as the most significant regulators, with IL10RA having a negative z-score and IFNG a positive z-score (Fig 5c). Conditional deletion of *Il10ra* in MNP (*Cx3cr1-IL10ra*^*Δ*^) and *Il10rb*^*-/-*^ mice [39, 40] leads to development of spontaneous colitis, similar to that observed in *Runx3*^**Δ**^ mice.

**Table 1:** Pro- and anti-inflammatory DEGs in Runx3^Δ^ MNP.

*Pro-inflammatory*				*Anti-inflammatory*			
DEGs in Runx3^Δ^ RM	DEGs in Runx3^Δ^ CD11b^+^ DC	Up in Il10R^Δ^ RM	Up in P4/P1	DEGs in Runx3^Δ^ RM	DEGs in Runx3^Δ^ CD11b^+^ DC	Up in Il10R^Δ^ RM	Up in P4/P1
***Acsl1***		**Yes**		***Adora2***		**Yes**	
***Ass1***	**Yes**	**Yes**		***Arg1***			
***Batf2***	**Yes**	**Yes**		*Ch25h*			**Yes**
***C3***	**Yes**	**Yes**		*Cited2*			
***Clec12a***		**Yes**		*Fblim1*			**Yes**
***Cxcl9***	**Yes**	**Yes**		***Gpx2***	**Yes**		
***Gzma***				*Gpx3*			
***Hif1a***				*Hpgds*			**Yes**
*Il1r1*	Yes			*Ifit3*			
***Il12a***	**Yes**	**Yes**		*Ifnb1*	Yes		**Yes**
***Inhba***		**Yes**		*Il10*			**Yes**
***Irg1***		**Yes**		*Il22ra2*			**Yes**
***Mif***	**Yes**			*Kmo*			**Yes**
***Nlrc5***	**Yes**	**Yes**		***Prdx5***			
***Nos2***		**Yes**		***Phlpp1***			
***Pdcd1lg2***	**Yes**	**Yes**		*Rcan1*	Yes		**Yes**
***Serpinb9***		**Yes**		*Sh2d1b1*			**Yes**
***Stat1***	**Yes**	**Yes**		*Siglech*			**Yes**
***Tarm1***		**Yes**		*Timd4*			**Yes**
*Tnf*			Yes				
***Tspan33***							

Pro- and anti-inflammatory DEGs in Runx3^Δ^ RM and CD11b^+^ DC and their up-regulation in Il10r^Δ^ and P4 RM. Gene names in bold and plain letters indicate up-regulated and down-regulated genes, respectively.

To examine a possible relationship between Runx3 and Il10 signaling in RM, we cross analyzed Runx3^**Δ**^ DEGs with transcriptional profile of Il10ra^Δ^ and Il10rb^-/-^ RM. Remarkably, 23 out of the 72 (32%) Runx3^**Δ**^ RM up-regulated genes were also up-regulated in the two other data sets and 40–50% of Runx3^**Δ**^ RM up-regulated genes overlapped with each of the Il10r^Δ^ RM data sets (Fig 5d). Moreover, common up-regulated genes in Runx3^**Δ**^, Cx3cr1-Il10ra^Δ^ and Il10rb^*-/-*^ RM included 15 of the 21 (71%) pro-inflammatory genes underscored above (Table 1). While MNP-*Runx3*^**Δ**^ and MNP-*Il10R*^**Δ**^ mice displayed a similar spontaneous colitis phenotype with a significant overlap of RM up-regulated genes, expression of *Il10ra* and *Il10rb* was unaffected in Runx3^**Δ**^ RM. Together, these observations led us to hypothesize that Runx3 is involved in MNP transcriptional regulation downstream of Il10-induced signaling.

Newly arrived colonic monocytes, termed P1 monocytes, differentiate through stages P2 and P3 until ultimately becoming mature P4 RM [37]. This process is accompanied by expression of 613 genes, in specifically P4 compared to P1 monocytes [37]. As Runx3 is expressed in colonic RM and in its absence the P4 mature stage is impaired, we hypothesized that Runx3 is involved in the maturation of colon P4 RM. As mentioned above, 128 down-regulated DEGs were identified by cross analysis of Runx3^**Δ**^/WT RM gene expression profiles (Fig 5a and S1 Table sheet 1). Intersecting these 128 RM Runx3^**Δ**^ down-regulated genes with the 613 P4-specific genes revealed a marked overlap of 58 genes, comprising 45% of Runx3^**Δ**^ down- regulated genes (Fig 5e). Remarkably, 11 of the 14 (79%) down-regulated anti-inflammatory genes in Runx3^**Δ**^ RM, including *Il10*, were among these 58 common genes (Table 1 and Fig 5e). Additionally, among these 58 down-regulated genes several were associated with the Notch pathway and cholesterol uptake. Maturation into colon P4 RM stage is dependent on up-regulation of various TGFβ signaling genes [36, 37]. As Runx3 has an established role in mediating TGFβ signaling [15], it is reasonable to speculate that the 58 down-regulated genes include Runx3-responsive TGFβ—regulated genes. Cross analysis of the P4-specific RM gene list with those that were down-regulated in *Tgfbr1*^Δ^*/RAG1*^*-/-*^ [37], revealed 62 common genes (S1 Table sheet 3). Intersecting these 62 genes with the above mentioned 58 P4-specific genes that were down-regulated in Runx3^**Δ**^ RM, revealed 9 common genes (Fig 5e and S1 Table sheet 3).

To gain further insight into potential Runx3 directly targeted genes, the list of 200 DEGs in Runx3^**Δ**^ RM (S1 Table sheet 1) was cross analyzed with lists of genes containing the hallmarks of transcriptionally active regions: ATAC-seq, H3K4me1 and H3K27ac ChIP-seq peaks in colonic RM [30]. Of particular relevance to our findings was the observation that a RUNX motif is highly enriched in enhancer regions specifically in intestinal macrophages as compared with macrophages from all other tissues [30]. Interestingly, this analysis revealed that out of these 200 DEGs, 120 (60%) harbored all three peak categories and 28 additional DEGs harbored both H3K4me1 and H3K27Ac peaks (S5a Fig), raising the number of peak-bearing DEGs to 148 (75%). Accordingly, only 25 DEGs (13%) contained none of these peaks (S5a Fig). Moreover, 133 of these 148 peak-harboring DEGs, including the 40 pro- and anti-inflammatory genes, contained at least one region in which at least two of the three above mentioned peak categories overlapped (in most cases H3K4me1 and H3K27ac). Statistical analysis using contingency tables revealed that the overlapping peaks were significantly enriched in DEGs compared to non-DEGs (Pearson Chi-squared test with Yate’s correction, X-squared 7.3992, df = 1, p-value 0.00652; Fisher’s exact test, p = 0.005033). Analysis of all regions encompassing overlapping H3K4me1 and H3K27ac peaks revealed that 118 DEGs associated with these peaks harbored a RUNX motif (S1 Table sheet 2 and see *Ass1* and *Il10* as examples in S5b Fig). Ten additional DEGs associated with overlapping ATAC and H3K27Ac peaks also harbored a RUNX motif, bringing the total number of DEGs with overlapping peaks containing RUNX to 128 (S1 Table sheet 2). The same statistical analysis revealed that overlapping peaks containing a RUNX motif were also enriched in DEGs compared to non-DEGs (Pearson Chi-squared test with Yate’s correction, X-squared 8.825, df = 1, p-value 0.00295; Fisher’s exact test, p = 0.002654). We defined these 128 Runx3^**Δ**^ RM DEGs as high-confidence Runx3 targets.

Interestingly, the human homologs of eight of these RM Runx3 high-confidence target genes, *CD300LF*, *CFB*, *IFIH1*, *IL10*, *NOS2*, *PLAU*, *PRDX5 and TNF*, harbored SNPs that are susceptibility loci for IBD, Crohn’s disease (CD) and/or ulcerative colitis (UC) (S1 Table sheet 2). Furthermore, we also noted that six out of the nine (Fig 5e) Runx3 putative targets regulated by TGF-β in RM P4 population harbor a RUNX-SMAD module in their Runx3 bound regions (four of these are shown in S5c Fig). Overall, the results suggest that Runx3 is involved in positively regulating TGFβ—dependent RM maturation and an Il10-driven anti-inflammatory response while suppressing a pro-inflammatory program.

### Runx3 positively regulates colonic CD11b^+^ DC differentiation genes

The colonic CD11b^+^ cDC2 subsets consist of double positive CD103^+^CD11b^+^ DC and their precursors CD103^-^CD11b^+^ DC. Both DC subsets express Runx3 (Fig 2e). The increased prevalence of colonic LP CD103^-^CD11b^+^ and the decrease in CD103^+^CD11b^+^ DC in *Runx3*^**Δ**^ compared to WT mice (Fig 3c) raised the possibility that, as in RM, Runx3 affects CD103^+^CD11b^+^ DC differentiation. Because it was hard to isolate sufficient numbers of colonic CD103^+^CD11b^+^ DC from *Runx3*^**Δ**^ mice, we compared the transcriptome of WT and Runx3^**Δ**^ colonic CD11b^+^ DC, including both CD103^-^CD11b^+^ and CD103^+^CD11b^+^ DC.

**Fig 6:**
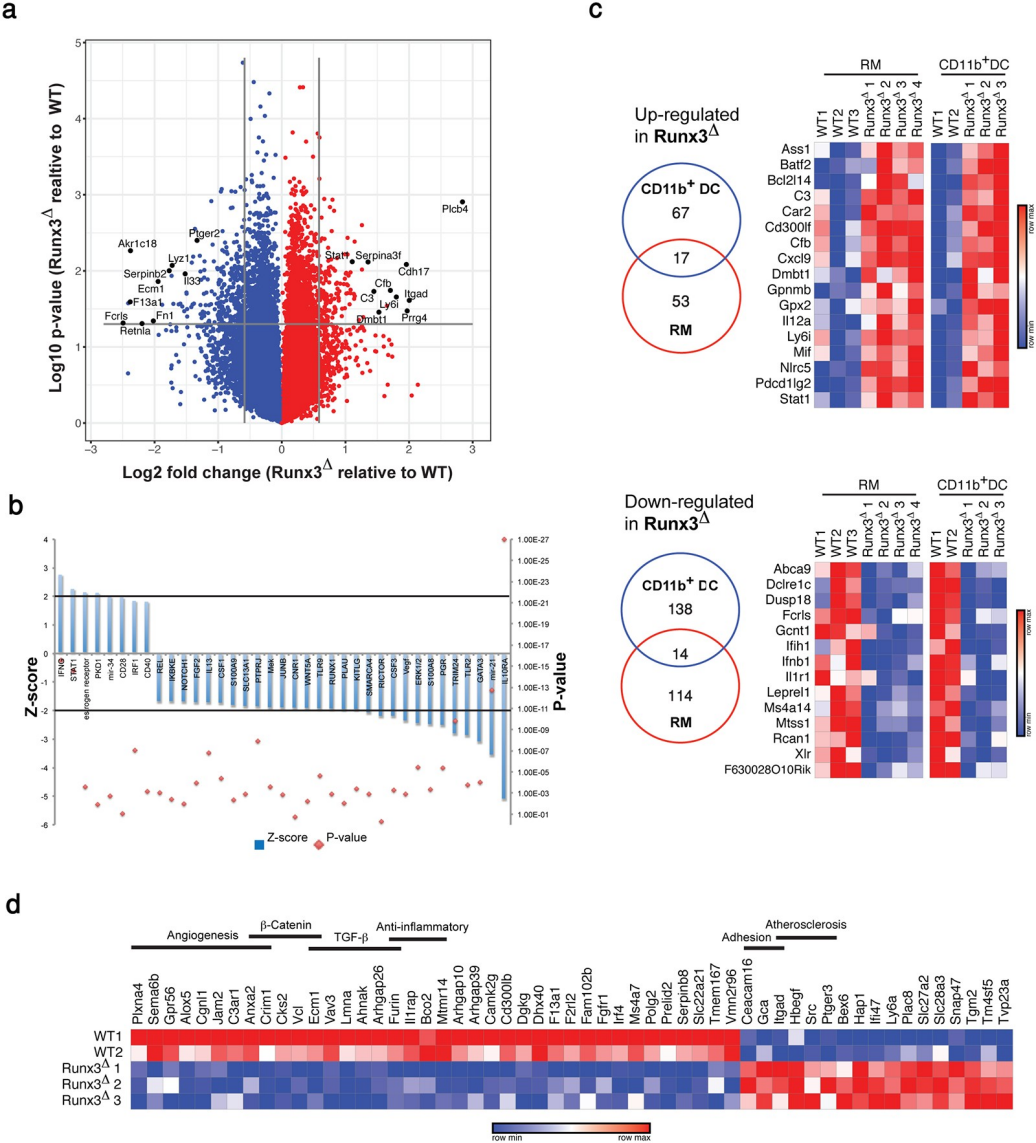
A fraction of DEGs between Runx3^Δ^ and WT CD11b^+^ DC are shared with Runx3^Δ^RM. (a) A Volcano plot of colonic CD11b^+^ DC DEGs in 6–8 weeks old *Runx3*^**Δ**^ and WT control mice. Numerous up- or down-regulated genes in Runx3^**Δ**^ CD11b^+^ DC are indicated. (b) GO analysis categorized by up-stream regulators. Cutoff and z-score values were set to 2. (c) Venn diagrams and heat maps showing common DEGs in colonic Runx3^**Δ**^ CD11b^+^ DC and RM. (d) Heat map showing standardized expression values of 65 high-confidence Runx3 target genes in CD11b^+^ DC.

The transcriptome profile revealed 84 up-regulated and 152 down-regulated genes (fold-change ≥ 1.5, p-value ≤ 0.05) in Runx3^**Δ**^ vs. WT CD11b^+^ DC (Fig 6a and S1 Table sheet 4). Similar to RM, GO analysis of DEGs in CD11b^+^ DC underscored IL10RA and IFNG as the top significant upstream regulators (Fig 6b). Importantly, we found a subset of 31 Runx3^**Δ**^ DEGs common to both RM and CD11b^+^ DC (Fig 6c), suggesting that some Runx3-regulated functions are shared between these two MNP populations. Remarkably, 13 of these 31 Runx3^**Δ**^ RM and CD11b^+^ DC common DEGs (42%) were inflammation-regulating genes, including 10 up-regulated pro-inflammatory and three down-regulated anti-inflammatory genes (Table 1). Moreover, most of the pro-inflammatory genes up-regulated in Runx3^**Δ**^ CD11b^+^ DC were shared by Il10ra and/or Il10rb-deficient RM (Table 1). These results suggest that Runx3^**Δ**^ CD11b^+^ DC display pro-inflammatory properties, similar to monocyte-derived CD11b^+^ DC following mild dextran sulfate sodium-induced colitis [24]. In addition, Runx3^**Δ**^ CD11b^+^ DC showed down-regulation of *Ifnb1* and IFN-β-regulated genes *Ifih1* and *Mx2* (Fig 6c and S1 Table sheet 4). Furthermore, enrichment of genes associated with β—catenin and TGF-β signaling was noted among Runx3^**Δ**^ CD11b^+^ DC down-regulated genes (Fig 6d).

Runx3 ChIP-seq analysis in the D1 DC cell line and splenic CD4^+^ DC [14] revealed 13,014 and 15,121 Runx3-bound regions (peaks), respectively, of which 6836 peaks overlapped, corresponding to 6422 genes. Interestingly, GREAT analysis using the “PANTHER Pathway” revealed that genes corresponding to overlapping Runx3-bound peaks in D1 cells and splenic CD4^+^ DC, as well as genes corresponding to peaks in colonic RM [30], were highly enriched for the term “Inflammation mediated by chemokine and cytokine signaling pathway” (S1 Table sheet 6). To determine the putative CD11b^+^ DC direct Runx3 target genes, we first cross-analyzed the list of DEGs in colonic Runx3^**Δ**^ CD11b^+^ DC with the list of common Runx3-bound genes in D1 and splenic CD4^+^CD11b^+^ DC. The results showed that 90 of the 236 DEGs in Runx3^**Δ**^ CD11b^+^ DC (38%) harbored Runx3-bound regions (S6a Fig). Moreover, 65 of these 90 Runx3-bound DEGs (71%) contained at least one region with a RUNX motif, suggesting that they are high-confidence Runx3-target genes (Fig 6d and S1 Table sheet 5). Moreover, 10 of the 31 common DEGs in Runx3^**Δ**^ CD11b^+^ DC and RM (32%) were also common high-confidence Runx3 target genes (Fig 6c and S6b Fig). Interestingly, three of these 10 common Runx3 target genes in colonic RM and CD11b^+^ DC (*Ifnb1*, *Pdcd1lg2* and *Stat1*) were either pro- or anti- inflammatory genes (S6c Fig). This observation suggested that they might contribute substantially to the *Runx3*^**Δ**^ mice colitis phenotype. Of note, the human homologs of four of the CD11b^+^ DC Runx3 targets, *CD300LF*, *IFIH1*, *IRF4* and *SLC22A5* (mouse *Slc22a21*) harbored SNPs associated with IBD, CD, UC and/or celiac disease (S1 Table sheet 5) and the two former genes are common Runx3 targets in RM and CD11b^+^ DC. Overall, the results indicated that Runx3 is important for the maturation of CD11b^+^ DC into anti-inflammatory and tolerogenic DC. They also indicated that loss of Runx3 in CD11b^+^ DC affects these anti-inflammatory and tolerogenic properties in a remarkably similar way to that found in Runx3^**Δ**^ RM and Il10r-deficient RM.

### Loss of Runx3 in MNP induces tolerogenic to inflammatory CD4 T cell transition

**Fig 7:**
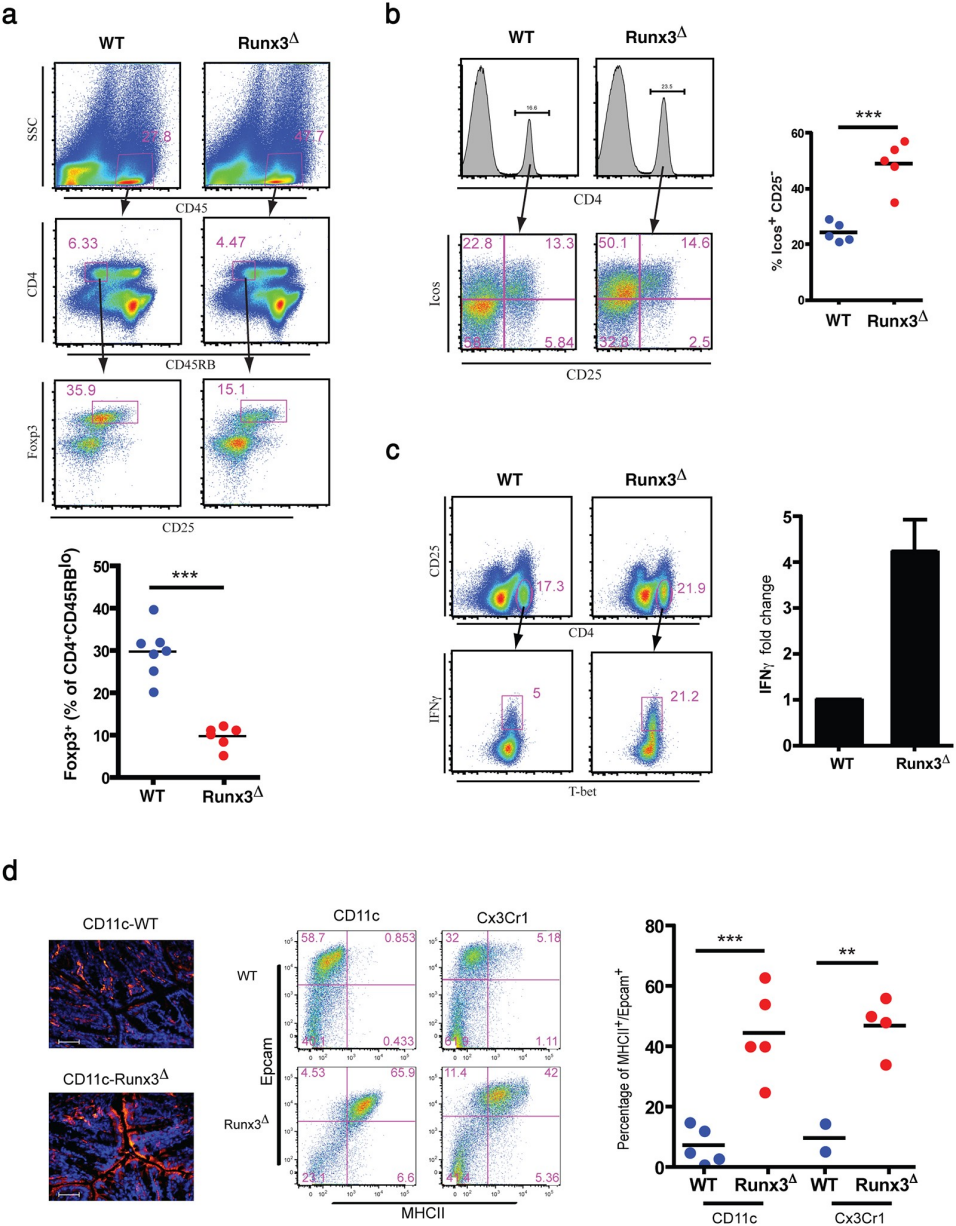
Runx3-deficient MNP cause a tolerogenic to inflammatory switch in colonic CD4^+^ T cells. (a) Analysis of Tregs in *Runx3*^**Δ**^ colonic LP. Representative flow cytometry analysis (top) and graphical summary (bottom) of CD25^+^Foxp3^+^ Tregs in colonic LP of WT *MNP-Runx3*^**Δ**^ mice. (b) Representative flow cytometry (left) and graphical summary (right) of inducible T cell costimulator (ICOS)-expressing WT and Runx3^**Δ**^ colonic LP CD4^+^CD25^-^ T cells. (c) Intracellular staining of IFN-γ in WT and Runx3^**Δ**^ colonic LP CD4^+^CD25^-^ T cells. (d) MHCII immunofluorescence (IF) staining in WT and *Runx3*^**Δ**^ colonic epithelia. Frozen sections from WT and *Runx3*^**Δ**^ colons stained with anti-MHCII. Magnification X40; scale bars 50μm (left). Flow cytometry analysis (middle) and graphical summary (right) of colonic epithelial cells from WT, *Runx3*^**Δ**^ and *Cx3cr1-Runx3*^*Δ*^ mice stained with anti-EpCAM and anti-MHCII. Dot plots horizontal bars represent mean values, unpaired two-tailed t-test **P<0.01, ***P<0.001.

The MNP switch from an anti- to pro-inflammatory response in the absence of Runx3 indicated an impaired activation of T cells. Altered expression of regulatory T cells (Tregs) inducing genes further supported this possibility. The down-regulated gene *Il33* in cDC2, encodes an important cytokine for intestinal Tregs generation [41]. Of potential relationship, *Il6* an additional down-regulated gene in cDC2 cells, induces generation of functional IL-10-producing T cells involved in suppressing inflammation in lung and colon [42], (S1 Table sheet 4). Furthermore, a previous study has shown that *Ifnb1*, an additional cDC2 down-regulated gene encoding IFN-β, is important for intestinal control of Tregs differentiation [43]. To verify whether Tregs were affected, we compared colonic Foxp3^+^ Tregs of WT and *Runx3*^**Δ**^ mice. The prevalence of Tregs was determined by gating on CD45^+^CD4^+^CD45RB^lo^ cells, followed by gating on CD25^+^Foxp3^+^ cells. Strikingly, Runx3^**Δ**^ colon LP showed a substantial reduction in the frequency of Foxp3^+^ Tregs (Fig 7a).

The inducible T cell co-stimulator (ICOS) plays a role in modulating different adaptive immune responses. Hence, we examined whether ICOS expression is affected in CD4^+^ lymphocytes of *Runx3*^**Δ**^ mice. Interestingly, we found that abundance of ICOS expressing cells in CD4^+^ lymphocytes of *Runx3*^**Δ**^ mice is significantly increased compared to those of WT mice (Fig 7b), suggesting an increase in number of activated CD4^+^ T cells in *Runx3*^**Δ**^ mice. GO analysis results suggested that Runx3^**Δ**^ MNP might also induce T-cell activation manifested by IFN-γ production. To examine the impact of Runx3 deletion in MNP on lymphocyte activation we analyzed IFN-γ expression in LP CD4^+^ T cells treated with 12-O-tetradecanoylphorbol-13-acetate (TPA) and ionomycin. The results indicated an increased abundance of IFN-γ expressing activated LP CD4^+^ T-cells in *Runx3*^**Δ**^ mice (Fig 7c).

Mucosal immunity depends partly on cross talk between the immune system and the epithelium. For instance, IFN-γ produced by CD4^+^ T cells plays a role in ameliorating the development of colitis by induction of major histocompatibility complex II (MHCII) expression in epithelium [44, 45]. Induction of MHCII in mucosal epithelial cells has also been reported in patients with IBD [46]. Consequently, we addressed whether colonic epithelial MHCII is affected in *Runx3*^**Δ**^ mice. Our analysis revealed a profound adluminal MHCII expression in Runx3^**Δ**^ LP epithelial cells, whereas MHCII expression in the WT LP was confined to leukocytes (Fig 7d left). Furthermore, we detected MHCII expression in colonic epithelium of both *Runx3*^**Δ**^ and *Cx3cr1-Runx3*^*Δ*^ mice, as evidenced by co-expression of MHCII and the epithelial marker EpCAM (Fig 7d middle and right). Given these findings, we speculate that the up-regulated expression of MHCII marks an epithelial involvement in the early inflammatory response in the Runx3-cKO, likely a downstream effect induced by the pro-inflammatory Runx3^**Δ**^ MNP. Collectively, the results imply that Runx3 expression in colonic LP MNP plays an important role in promoting intestinal immunological tolerance, via maintenance of LP Foxp3^+^ Tregs and significantly inhibiting IFN-γ production by CD4^+^ T-cells. The diversion from regulatory into pro-inflammatory CD4^+^ T cells in Runx3^**Δ**^ colon strengthen the impact of transcriptional transition of Runx3^**Δ**^ MNP into an immature pro-inflammatory state.

## Discussion

The large surface area of intestinal mucosa exposed to external environment poses a great challenge to mucosal immune system to maintain a balance between defense against pathogens and tolerance to commensal bacteria and food antigens. Disrupting this balance can leads to IBD [47]. More than 200 human IBD susceptibility loci have been identified, mostly in genomic regions in the vicinity of genes expressed in various immune cells, including those on chromosome 1p36 in the *RUNX3* locus [2, 48–50]. Moreover, many IBD susceptibility loci have been associated with genes that are RUNX3 targets in various immune cells [2]. Previously we reported that Runx3^-/-^ mice develop early onset spontaneous colitis [17]. The fact that Runx3 is not expressed in normal colonic epithelium [17, 18], suggests that loss of a leukocytic cell-autonomous Runx3 function was the driving force of colitis development in these mice [17]. Here, we provide direct evidence that transfer of Runx3^-/-^, but not WT, FL, or BM cells into lethally irradiated mice induced colitis in the recipient mice. Moreover, conditional deletion of *Runx3* in MNP, but not in lymphocytes, recapitulated the spontaneous development of colitis observed in *Runx3*^*-/-*^ mice. These results indicate that Runx3 expression in MNP is important for their known role in maintaining GIT homeostasis [34].

The loss of Runx3 in MNP results in a decreased abundance of colonic P3-P4 mature anti-inflammatory RM and CD103^+^CD11b^+^ cDC2, which occurs prior to the onset of significant colitis symptoms, suggesting that these changes are indeed the cause of colitis. This premise is supported by the finding that Runx3^**Δ**^ BM transfer to lethally irradiated mice induced the same changes in MNP populations as well as colitis, whereas mice transplanted with an equal number of WT and Runx3^**Δ**^ BM cells showed a normal MNP balance and remained healthy. These data are consistent with the conclusion that presence of WT BM cells overcomes the colitogenic effect of the transplanted Runx3^**Δ**^ BM cells.

Analysis of Runx3^**Δ**^ and WT colonic RM transcriptomes, revealed a prominent up- and down-regulation of pro- and anti-inflammatory genes, respectively, in Runx3^**Δ**^ RM. These results strongly indicate that loss of Runx3 in RM induces an anti- to pro-inflammatory switch in their biological properties. Comparison of RM DEGs in Runx3^**Δ**^ with those in Il10ra^**Δ**^ and Il10rb^-/-^, which also induce colitis [39, 40], revealed a gain of pro-inflammatory hallmarks in all three models of spontaneous colitis.

Loss of Runx3 in RM did not affect expression of *IL10ra* and *IL10rb* in our data, and neither was expression of *Runx3* significantly affected by loss of Il10rb [39]. Therefore, the similar spontaneous colitis phenotype in these three strains cannot be explained by cross-regulation between Runx3 and signals emanating from the Il10 receptors. However, it is possible that activation of Stat3 TF, downstream of Il10 receptor signaling [38)], collaborates with nuclear Runx3, which can explain the partially shared effect on gene expression when either of these TFs is deleted. This possibility is supported by the known ability of Stat and Runx proteins to physically interact with each other [51], and the spontaneous colitis induced in mice harboring Stat3-deficient MNP [52–54]. Of note, the fact that common DEGs in Runx3- and Ill0 receptor-deficient RM is mostly confined to up-regulated genes, suggests that collaboration between Runx3 and Stat3 is employed mainly to suppress their targets, particularly pro-inflammatory genes. It should also be emphasized that while Runx3 and Il10r deficiencies in RM lead to colitis, each model bears unique features. For example, down regulation of anti-inflammatory genes occurs in Runx3^**Δ**^ RM, but is not evident in the two Il10r models. Furthermore, a recent study reported that Il10ra^**Δ**^ RM show increased expression of Il23, which induces Il22 production in T cells leading to hypertrophy of colonic epithelium [55]. In contrast, no change was noted in *Il23* expression in Runx3^**Δ**^ RM, but expression of *Il22ra2*, encoding a very potent antagonist of Il22 receptor signaling, was down-regulated. Because the balance of Il22 and its antagonist Il22ra2 (IL22RB) is important for intestinal homeostasis [56], it is conceivable that reduced *Il22ra2* expression in Runx3^**Δ**^ RM, increases response to Il22 itself and could thus elicit an inflammatory response in the epithelium.

The differentiation of intestinal P1 monocytes to fully mature anti-inflammatory P4 RM is a TGFβ-dependent process [37] and we have now shown that P4 RM expressed a higher level of Runx3 compared to P1 monocytes. Loss of Runx3 in MNP results in defective RM differentiation associated with reduced expression of anti-inflammatory genes. The finding of reduced expression of TGFβ and Notch-regulated genes is in line with the defect of Runx3^**Δ**^ RM differentiation. Thus, Runx3 function in normal RM to repress pro-inflammatory genes and induce anti-inflammatory genes, is consistent with its ability to protect against colitis. Furthermore, 60% of all DEGs in Runx3^**Δ**^ RM, including most of the inflammatory genes, are in fact high-confidence Runx3-target genes as judged by their harboring overlapping ATAC and enhancer chromatin marks peaks containing a RUNX binding motif. Moreover, as with human *RUNX3* itself [2], human homologs of eight of these high-confidence Runx3 target genes in RM contain known susceptibility loci for IBD, CD, UC or celiac GIT diseases.

Runx3 is a key player in cell-lineage fate decisions, including in DC and do so to some extent by mediating response to TGFβ. For example, Runx3 regulates TGFβ-mediated lung DC functions, facilitates specification of murine splenic CD11b^+^Esam^hi^ DC and is mandatory to TGFβ-dependent development of skin Langerhans cells [14, 15]. TGFβ-dependence of differentiation of intestinal CD103^+^CD11b^+^ DC has also been demonstrated [35, 37]. Recent published study showed reduction in intestinal CD103^+^CD11b^+^ DC in mice ablated for Cbfb, the Runx protein-dimerizing partner, in DC. In contrast to our findings, these mice developed spontaneous colitis at relatively older age possibly due to usage of mixed background mice [57]. We found that Runx3 is expressed at low levels in ~ 20% of intestinal cDC1, whereas it is highly expressed in majority of cDC2 cells. Given that Runx3^**Δ**^ LP shows reduced abundance of these mature CD103^+^CD11b^+^ cDC2, it is conceivable that Runx3 participates in TGFβ signaling in cDC2 subsets. Mice lacking colonic cDC1 can still establish tolerance, presumably by their CD11b^+^ cDC2 subsets [58]. Thus, the fact that colonic CD11b^+^ cDC2 are affected in *Runx3*^**Δ**^ mice may imply that these cells contribute to colitis development.

Analysis of cDC2 transcriptomes of Runx3^**Δ**^ vs. WT mice revealed that merely 31 of the 236 DEGs in Runx3^**Δ**^ cDC2 were common with those of Runx3^**Δ**^ RM. Remarkably, ~40% of these 31 common DEGs were inflammation-regulating genes, including 10 commonly up-regulated pro-inflammatory genes and three commonly down-regulated anti-inflammatory genes. Most of these common pro-inflammatory genes that are up-regulated in Runx3^**Δ**^ RM and cDC2 are also up-regulated in Il10ra and/or Il10rb-deficient RM (Table 1). These results imply that like their Runx3^**Δ**^ RM counterparts, LP Runx3^**Δ**^ cDC2 contribute to the colitis phenotype by acquiring a pro-inflammatory state. Cross-analysis of Runx3^**Δ**^ cDC2 DEGs with genes that harbored overlapping Runx3-bound peaks in ChIP-seq assays revealed that 65 of these genes contained a RUNX motif, marking them as high-confidence Runx3 target genes. Ten of these cDC2 high-confidence Runx3 targets were common to those in RM and human homologs of four, *CD300LF*, *IFIH1*, *IRF4* and *SLC22A5* (mouse *Slc22a21*) harbored SNPs associated with IBD, CD, UC and/or celiac disease. Of particular interest is the known importance of Irf4 for the survival of intestinal CD103^+^CD11b^+^ DC [59], which can explain the reduced abundance of these cells in Runx3^**Δ**^ colon.

Intestinal DCs participate in immune tolerance and barrier protection by driving differentiation of Treg cells and Th17 cells, respectively [60]. Interestingly, some of the DEGs among Runx3^**Δ**^ cDC2 suggest an impairment of β-catenin signaling pathway, which is important in induction of tolerogenic DC [36, 37]. Another critical element in the tolerance breach in Runx3^**Δ**^ mice is the substantial reduction in number of Foxp3^+^ Treg cells, a crucial component in inducing GIT mucosal tolerance. This reduction can be attributed to a defective ability of Runx3^**Δ**^ cDC2 to generate Treg cells, as essential Treg-inducing factor genes including *Aldh1a2*, *Il33* and *Ifnβ* [41, 43, 61] are down-regulated in Runx3^**Δ**^ cDC2. The decreased *Ifnβ* expression that is also evident in Runx3^**Δ**^ RM suggests that loss of Runx3 expression in RM participates in the failure of Runx3^**Δ**^ mice to generate and/or maintain GIT Tregs. In addition, induced surface expression of Pd-l2 in Runx3^**Δ**^ MNP exemplifies the pro-inflammatory state acquired by Runx3^**Δ**^ RM, which can explain the increased production of IFNγ by Runx3^**Δ**^ CD4^+^ T-cells.

Besides similarities in inflammatory DEGs between Runx3^**Δ**^ RM and cDC2, other DEGs unique to each MNP subset may also contribute to the colitis phenotype. RM are normally maintained in a state of inflammatory anergy through acquisition of a non-inflammatory gene expression profile [36, 62, 63], yet they retain their bactericidal capacity [64]. Interestingly, the anti-bacterial autophagy gene *Clec12a*, which is reportedly associated with an increased risk for CD [65] and *Slc7a11*, a potential blocking target for treatment of IBD patients [66], were up-regulated specifically in Runx3^**Δ**^ RM but not in Runx3^**Δ**^ cDC2.

While the increased and decreased expression of pro-inflammatory and anti-inflammatory genes, respectively, in Runx3^**Δ**^ MNP can contribute to colitis development, it may be insufficient, as in the context of the BM chimera replenishment assays, mixed WT/Runx3^**Δ**^ MNP chimeric mice were protected from colitis. This finding implies that WT Runx3-sufficient MNP confer an immune-suppressive GIT condition, by maintaining a proper balance of pro- and anti-inflammatory gene expression in MNP themselves together with an indirect effect that prevents the loss of Tregs.

To summarize, all of our results point toward one major conclusion: MNP Runx3 maintains colon homeostasis by directing proper colon MNP specification into mature anti-inflammatory MNP and concomitantly repressing expression of a harmful pro-inflammatory program, similar to that which occurs in Il10 receptor-deficient MNP. Another layer of MNP Runx3 contribution to intestinal homeostasis is by its impact on maintaining colonic Tregs. These results imply that human MNP RUNX3 plays an important role in preventing development of inflammatory GIT diseases in humans, including IBD, CD, UC, and celiac disease. This premise is strongly supported by the presence of susceptibility loci for these diseases in the RUNX3 gene itself and in 10 other genes that are high-confidence RUNX3 targets in RM and/or cDC2.

## Supporting information

S1 Fig Runx3 expression in colon MNP. (a) Flow cytometry analysis of Runx3-GFP expression in LP RM and monocytes. (b) Flow cytometry analysis of Runx3-GFP expression in the two circulating blood monocyte subsets. (c) Flow cytometry analysis of Runx3-P1^AFP/+^ (blue) or Runx3-P2^GFP/+^ (green) expression in LP RM and CD103^+^CD11b^+^ DC relative to WT control (red). Representative experiment (out of three with similar results) is shown.
(PDF)

S2 Fig Imbalance of colon MNP subsets in *Runx3*^Δ^ mice. (a) Graphical summary of total number of Cx3cr1^Int^ and Cx3cr1^Hi^ CD11b^+^ RM in *Runx3*^**Δ**^-*Cx3cr1*^GFP/+^ and WT-*Cx3cr1*^GFP/+^. (b) Graphical summary comparison of DC subsets prevalence and cell subsets number between *Runx3*^**Δ**^ and WT at 5–6 weeks (top). Representative flow cytometry analysis (out of three with similar results) comparing CD24a expression in CD103^+^CD11b^+^ DC. Note the reduced CD24a expression in *Runx3*^**Δ**^ (middle). Graphical summary comparing CD103^+^CD11b^+^CD24a^+^ cell number and CD24 expression level between *Runx3*^**Δ**^ and WT in 6-week-old mice. Note the reduced CD24a expression in *Runx3*^**Δ**^ (bottom). (c) Representative flow cytometry analysis (out of three with similar results) comparing CD103 expression in CD11b^+^ DC. *Runx3*^**Δ**^ and WT mice analyzed at 6-weeks of age. Note the reduced CD103 expression in *Runx3*^**Δ**^ (middle). Graphical summary comparing CD103 expression level in CD11b^+^ DC between Runx3^**Δ**^ and WT (right). Dot plots horizontal bars represent mean values, unpaired two-tailed t-test * p<0.05, **p<0.01.
(PDF)

S3 Fig Irradiated mice reconstituted with WT, *Runx3*^Δ^ or a mixture of WT/*Runx3*^Δ^ BM Cells. (a) Body weight follow-up of Runx3^**Δ**^, mixed chimera and WT BM recipient mice. (b) Abundance of RM, CD11b^+^ DC and Ly6c^+^ monocytes in Runx3^**Δ**^, mixed chimera and WT BM recipient mice. Dot plots horizontal bars represent mean values, unpaired two-tailed t-test * p<0.05, **p<0.01.
(PDF)

S4 Fig WT versus *Runx3*^Δ^ colon MNP transcriptional profile comparison. (a) Sorting strategy of colonic LP RM (CD45^+^CD11c^+^MHCII^+^CD11b^+^F4/80^+^Ly6c^-^) and CD11b^+^ DC (CD45^+^CD11c^+^MHCII^+^CD11b^+^F4/80^-^Ly6c^-^) derived from 6-8-week old *Runx3*^**Δ**^ and WT littermate mice. Cells sorted from 3–4 mice were pooled for each sample. (b) Principal component analysis (PCA) analysis of all microarray samples, showing clear separation of RM and CD11b^+^ DC. (c) qPCR validation of DEGs between Runx3^**Δ**^ and WT RM in the microarray analysis. (d) Comparison of Pdcd1lg2 expression between Runx3^**Δ**^ and WT RM and CD11b^+^ DC. Flow cytometry (top) and graphical summary (bottom) of 3 biological repeats are shown. (e) Comparison of Clec12a expression between Runx3^**Δ**^ and WT RM and CD11b^+^DC. Flow cytometry (upper) and graphical summary (lower) of 3 biological repeats are shown. Unpaired two-tailed t-test *p<0.05.
(PDF)

S5 Fig Identification of high-confidence Runx3 regulated genes in colon RM. (a) Cross-analysis of DEGs in colonic LP Runx3^**Δ**^ RM with genes bearing ChIP-seq peaks in WT colonic RM. (b) UCSC genome browser display (mm9) of two high-confidence DEGs, *Il10* and *Ass1* (top and bottom, respectively), with peaks containing a RUNX motif in the boxed region. (c) UCSC genome browser display (mm9) of four TGF-β regulated high-confidence Runx3 target genes in RM. Peaks containing a RUNX and SMAD motifs are marked by boxed region. The DNA sequences demonstrate the RUNX (red)-SMAD (green) module.
(PDF)

S6 Fig Identification of high-confidence Runx3 regulated genes in colon cDC2. (a) Venn diagram depicting cross-analysis of DEGs in colonic Runx3^**Δ**^ CD11b^+^ DC with genes harboring Runx3 peaks in D1 cells and splenic CD4^+^ DC. (b) Venn diagram depicting cross-analysis of Runx3 target genes in CD11b^+^ DC and Runx3 target genes in colonic RM. (c) UCSC genome browser display (mm9) of D1 cells and splenic CD4^+^ DC Runx3 occupied regions in three high-confidence DEGs (*Ifnb1*, *Stat1* and *Pdcd1lg2*) common to colonic CD11b^+^ DC and RM.
(PDF)

S7 Fig Efficient conditional deletion of Runx3 in CD8^+^ T cells. Protein extract from mouse thymocytes or FACS-sorted splenic CD8^+^ T cells from WT or *Runx3*^*fl/fl*^:^*Lck-Cre*^ mice were loaded (10 μg/lane) on SDS-polyacrylamide gel and separated by electrphoresis. Proteins were blotted onto nitrocellulose membrane and blots reacted sequentially with in house anti-Runx1, anti-Runx3 and anti-Emerin antibodies, followed by peoxidase-conjugated anti-rabbit IgG secondary antibody. Signals were developed using the ECL kit (Amersham Pharmacia) and detected by exposure to an x-ray film.
(PDF)

S1 Table See a separate Excel file. Sheet 1, List of DEGs in Runx3^**Δ**^ versus WT colonic RM. Sheet 2, Putative Runx3 target genes in RM. Gene names marked in red indicate human susceptibility genes for GIT diseases. CD, Crohn’s disease; IBD, inflammatory bowel disease; UC, ulcerative colitis. Sheet 3, Venn diagram showing cross analysis of genes up-regulated expression in P4 mature colonic RM versus P1 monocytes (blue circle), down-regulated expression in TGFbR-cKO vs WT RM (yellow circle) and DEGs in Runx3^**Δ**^ versus WT colonic RM (green circle). The lists of common genes to these three circles is included. Sheet 4, List of DEGs in Runx3^**Δ**^ versus WT colonic CD11b^+^ DC. Sheet 5, List of putative Runx3 target genes in colonic CD11b^+^ DC, defined as DEGs in Runx3^**Δ**^ versus WT colonic CD11b^+^ DC that harbor overlapping Runx3-bound peaks in D1 and splenic CD4^+^ DC which contain RUNX motif. Gene names marked in red indicate human susceptibility genes for GIT diseases. Sheet 6, Panther Pathway enriched terms of H3K27ac-marked peaks in colonic RM (left) and overlapping Runx3-bound peaks in splenic CD4^+^ DC and D1 cells (right).
(XLSX)
