# Supplementary figures and images for: Runx3 prevents spontaneous colitis by directing the differentiation of anti-inflammatory mononuclear phagocytes
Source: PLoS One. 2020 May 26;15(5):e0233044. doi: 10.1371/journal.pone.0233044 (PMC7250423; doi:10.1371/journal.pone.0233044)

**a**

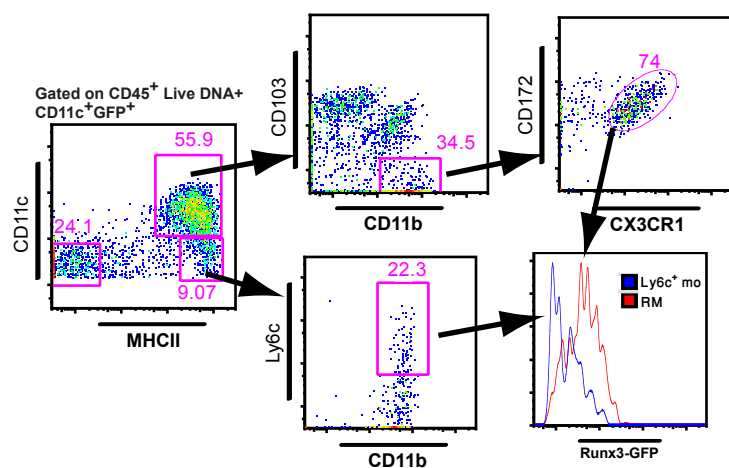

**b**

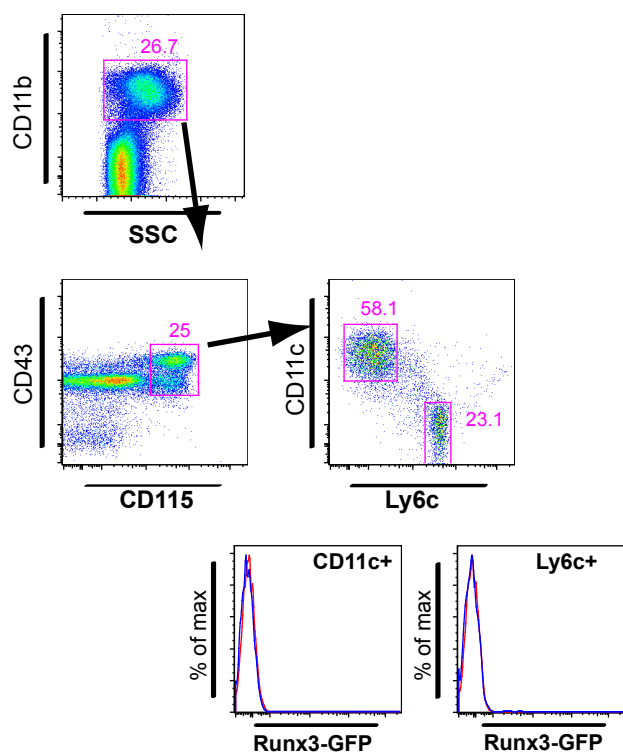

**C**

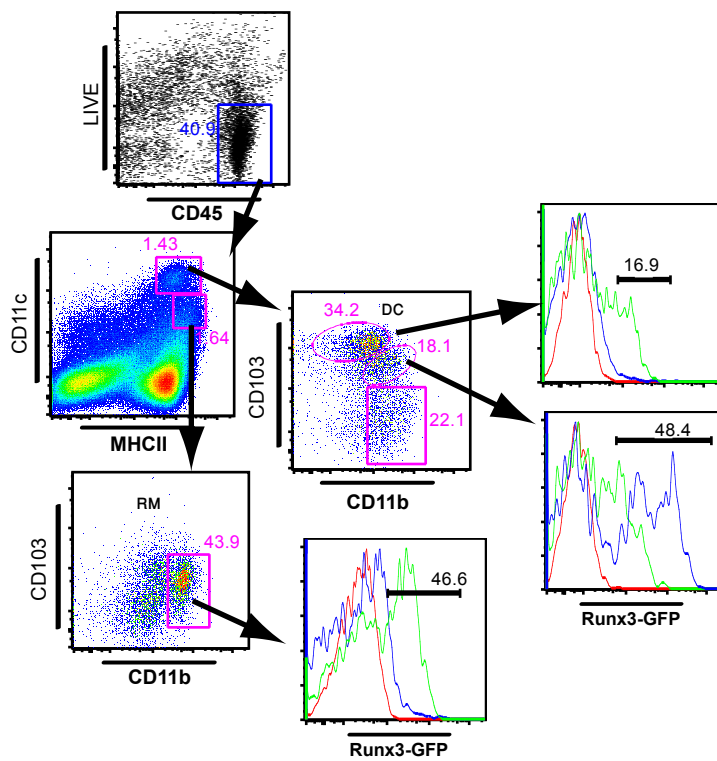

Supplement: S1 Fig — (a) Flow cytometry analysis of Runx3-GFP expression in LP RM and monocytes. (b) Flow cytometry analysis of Runx3-GFP expression in the two circulating blood monocyte subsets. (c) Flow cytometry analysis of Runx3-P1AFP/+ (blue) or Runx3-P2GFP/+ (green) expression in LP RM and CD103+CD11b+ DC relative to WT control (red). Representative experiment (out of three with similar results) is shown. (PDF) [file pone.0233044.s001.pdf]

a

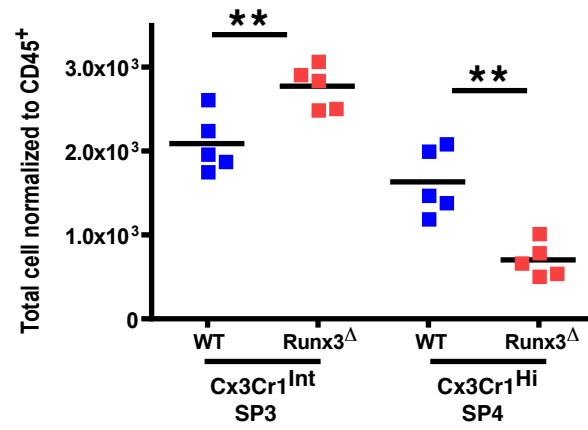

b

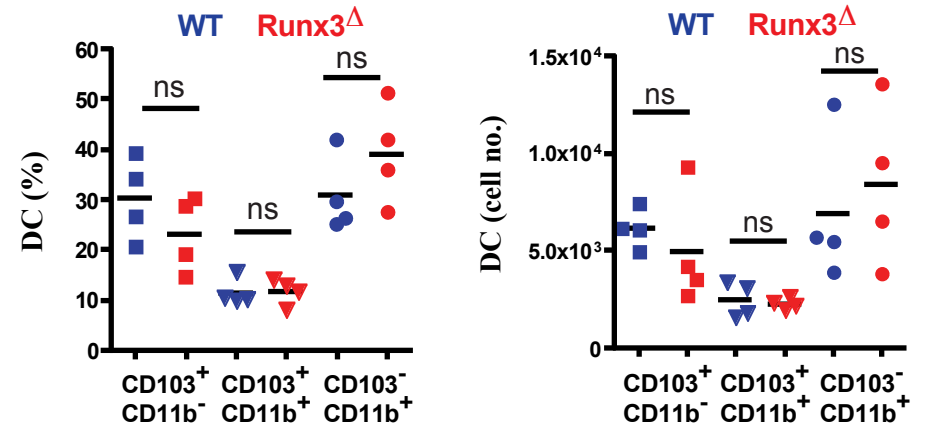

c

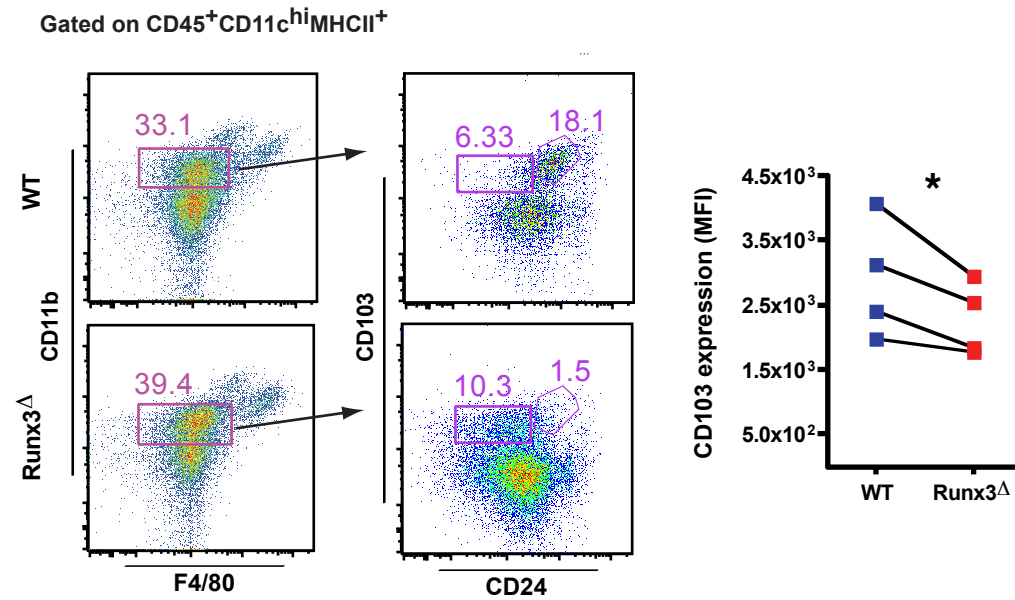Gated on CD45<sup>+</sup>CD11c<sup>+</sup>CD103<sup>+</sup>MHCII<sup>+</sup>F4/80<sup>-</sup>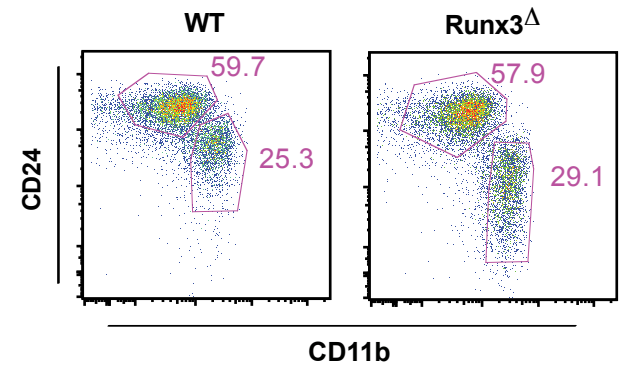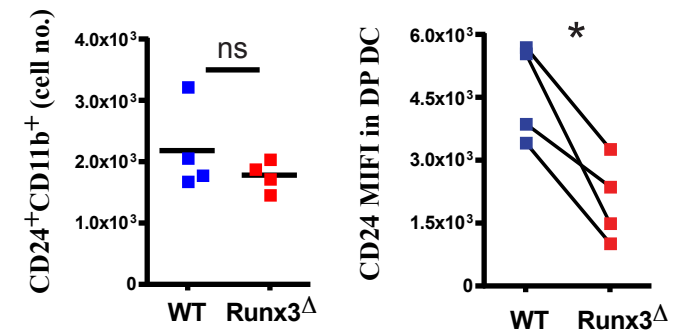

Supplement: S2 Fig — (a) Graphical summary of total number of Cx3cr1Int and Cx3cr1Hi CD11b+ RM in Runx3Δ-Cx3cr1GFP/+ and WT-Cx3cr1GFP/+. (b) Graphical summary comparison of DC subsets prevalence and cell subsets number between Runx3Δ and WT at 5–6 weeks (top). Representative flow cytometry analysis (out of three with similar results) comparing CD24a expression in CD103+CD11b+ DC. Note the reduced CD24a expression in Runx3Δ (middle). Graphical summary comparing CD103+CD11b+CD24a+ cell number and CD24 expression level between Runx3Δ and WT in 6-week-old mice. Note the reduced CD24a expression in Runx3Δ (bottom). (c) Representative flow cytometry analysis (out of three with similar results) comparing CD103 expression in CD11b+ DC. Runx3Δ and WT mice analyzed at 6-weeks of age. Note the reduced CD103 expression in Runx3Δ (middle). Graphical summary comparing CD103 expression level in CD11b+ DC between Runx3Δ and WT (right). Dot plots horizontal bars represent mean values, unpaired two-tailed t-test * p<0.05, **p<0.01. (PDF) [file pone.0233044.s002.pdf]

a

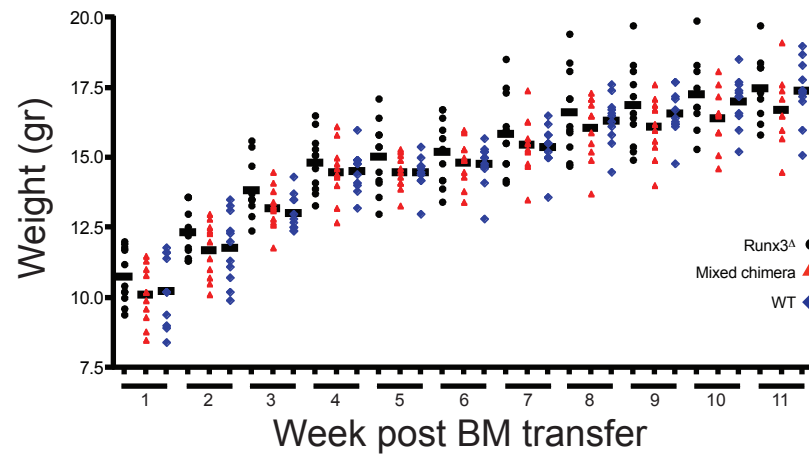

b

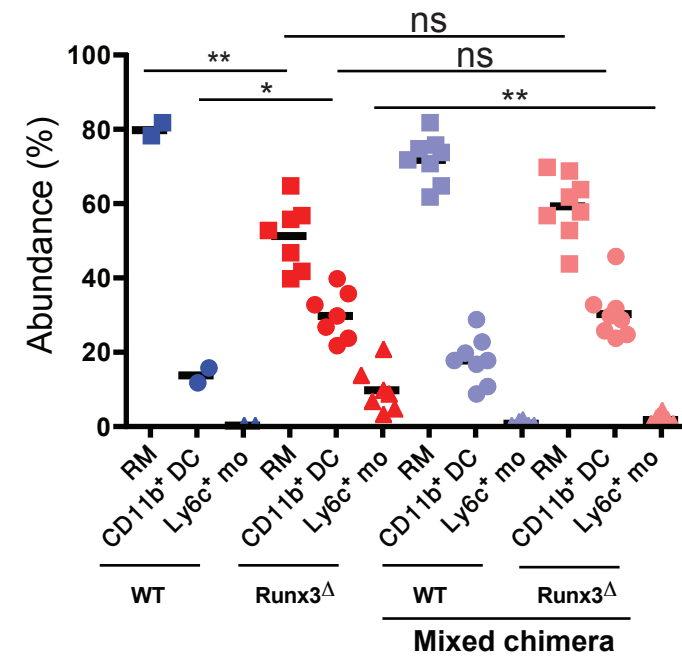

Supplement: S3 Fig — (a) Body weight follow-up of Runx3Δ, mixed chimera and WT BM recipient mice. (b) Abundance of RM, CD11b+ DC and Ly6c+ monocytes in Runx3Δ, mixed chimera and WT BM recipient mice. Dot plots horizontal bars represent mean values, unpaired two-tailed t-test * p<0.05, **p<0.01. (PDF) [file pone.0233044.s003.pdf]

a

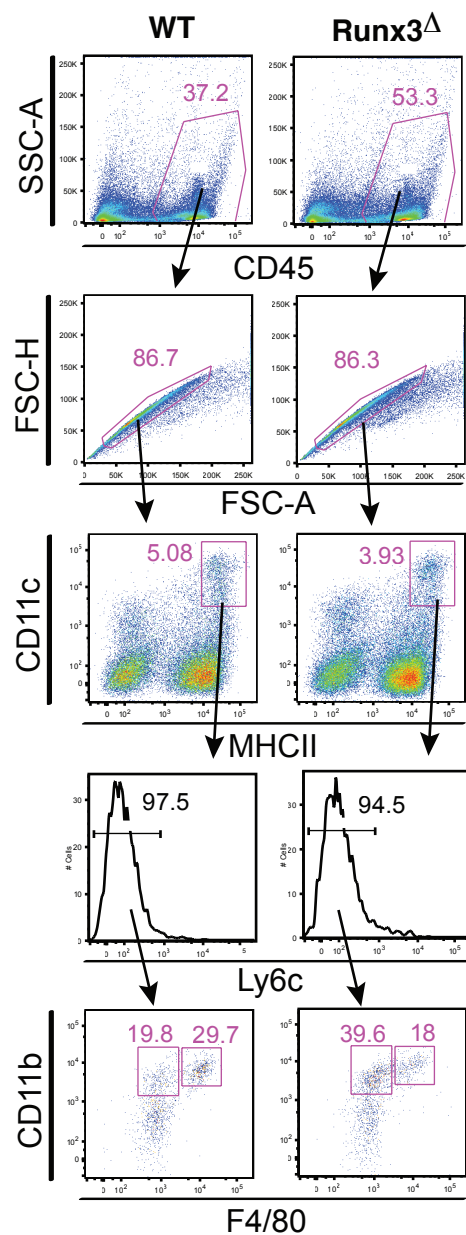

b

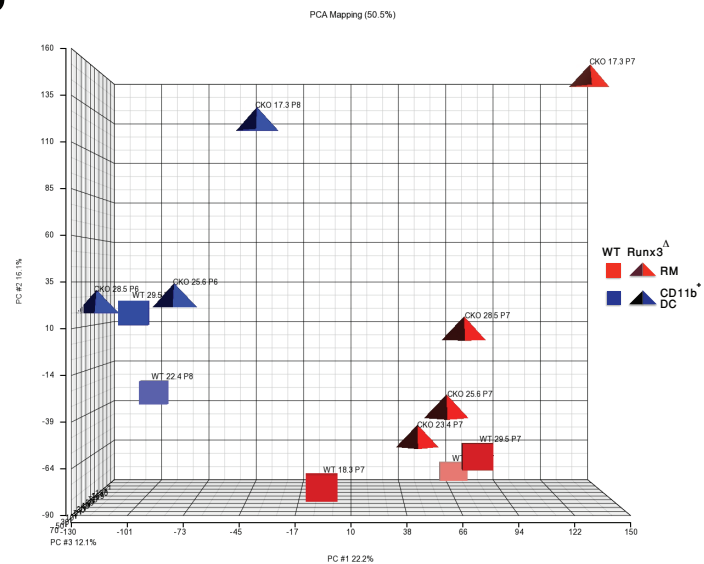

c

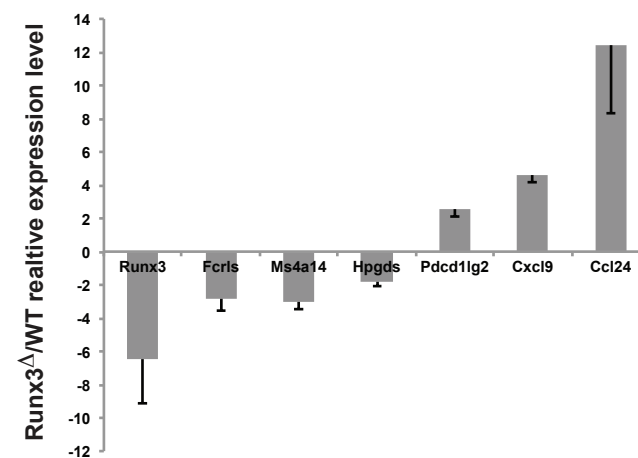

d

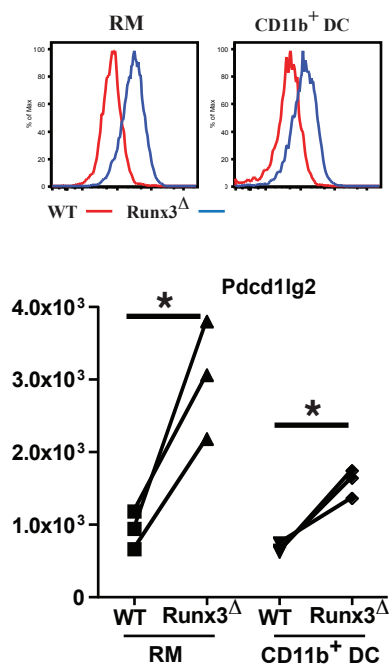

e

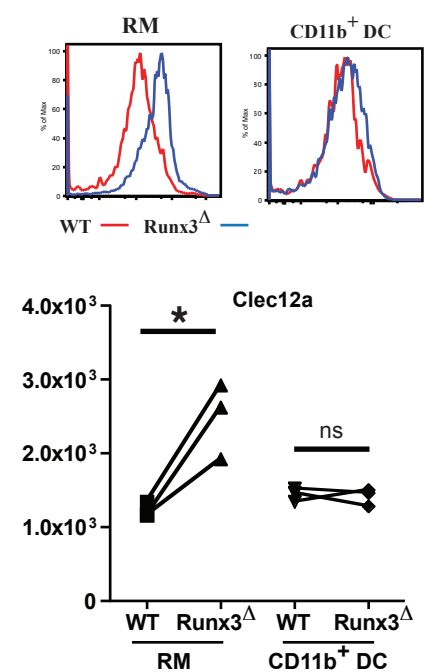

Supplement: S4 Fig — (a) Sorting strategy of colonic LP RM (CD45+CD11c+MHCII+CD11b+F4/80+Ly6c-) and CD11b+ DC (CD45+CD11c+MHCII+CD11b+F4/80-Ly6c-) derived from 6-8-week old Runx3Δ and WT littermate mice. Cells sorted from 3–4 mice were pooled for each sample. (b) Principal component analysis (PCA) analysis of all microarray samples, showing clear separation of RM and CD11b+ DC. (c) qPCR validation of DEGs between Runx3Δ and WT RM in the microarray analysis. (d) Comparison of Pdcd1lg2 expression between Runx3Δ and WT RM and CD11b+ DC. Flow cytometry (top) and graphical summary (bottom) of 3 biological repeats are shown. (e) Comparison of Clec12a expression between Runx3Δ and WT RM and CD11b+DC. Flow cytometry (upper) and graphical summary (lower) of 3 biological repeats are shown. Unpaired two-tailed t-test *p<0.05. (PDF) [file pone.0233044.s004.pdf]

a

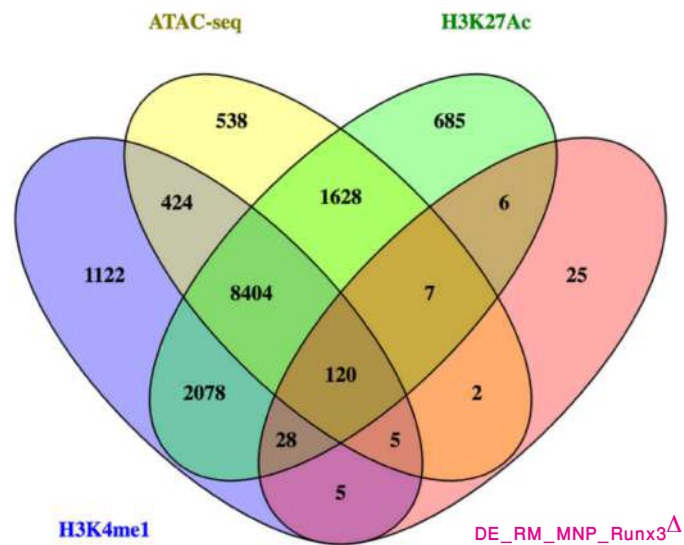

b

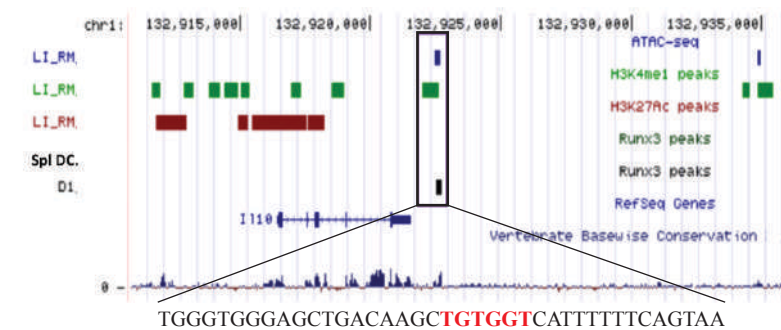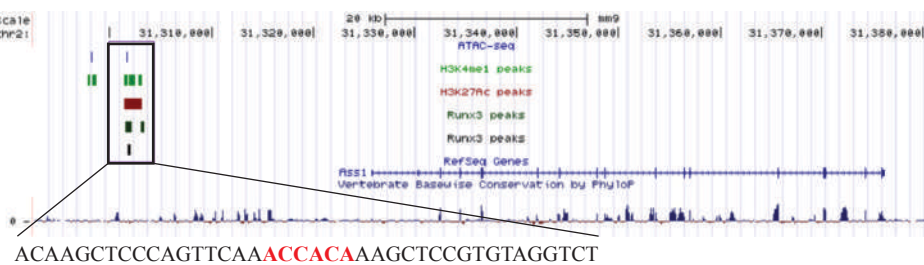

c

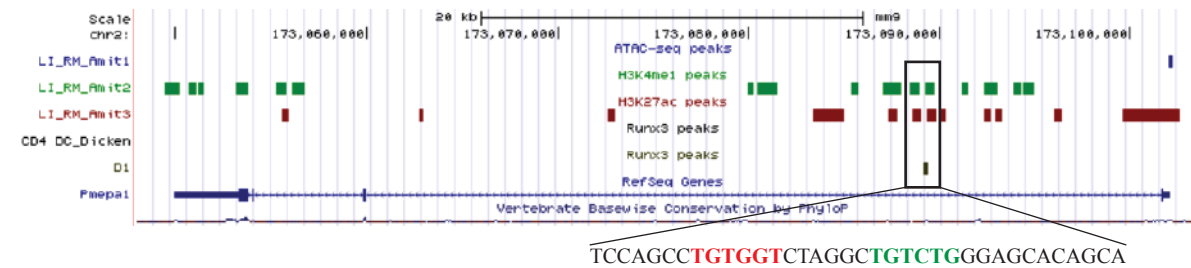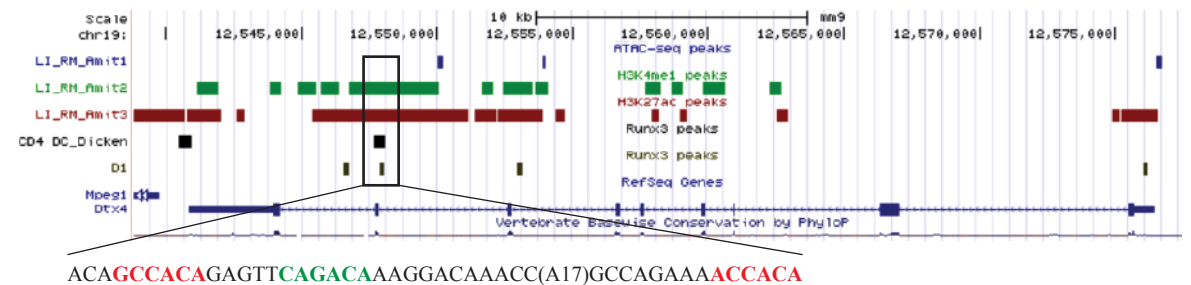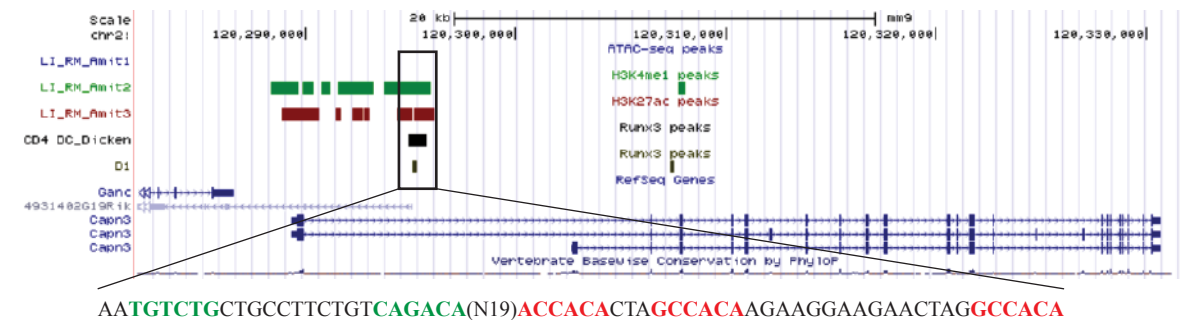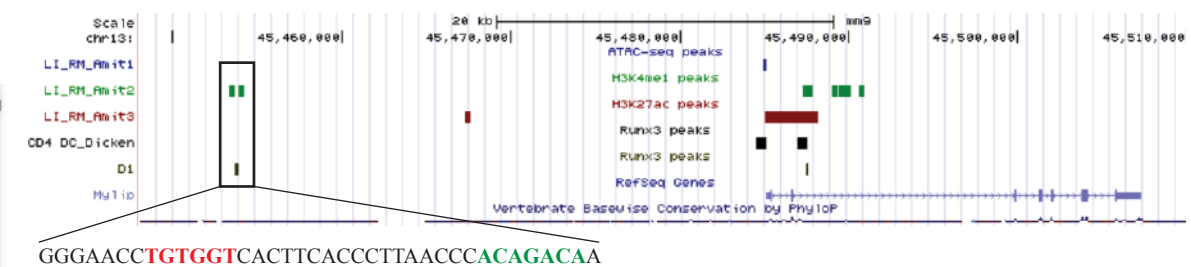

Supplement: S5 Fig — (a) Cross-analysis of DEGs in colonic LP Runx3Δ RM with genes bearing ChIP-seq peaks in WT colonic RM. (b) UCSC genome browser display (mm9) of two high-confidence DEGs, Il10 and Ass1 (top and bottom, respectively), with peaks containing a RUNX motif in the boxed region. (c) UCSC genome browser display (mm9) of four TGF-β regulated high-confidence Runx3 target genes in RM. Peaks containing a RUNX and SMAD motifs are marked by boxed region. The DNA sequences demonstrate the RUNX (red)-SMAD (green) module. (PDF) [file pone.0233044.s005.pdf]

a

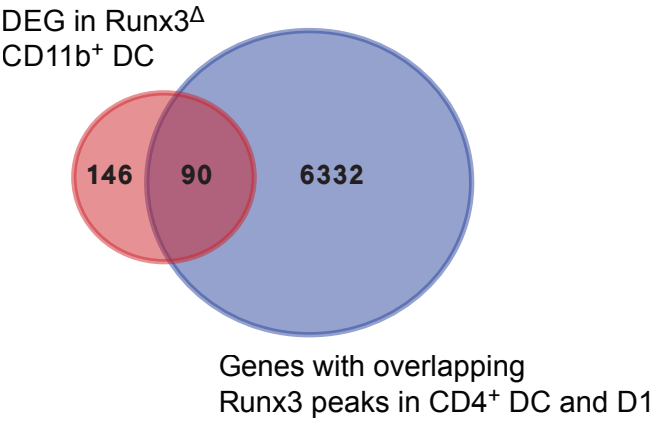

b

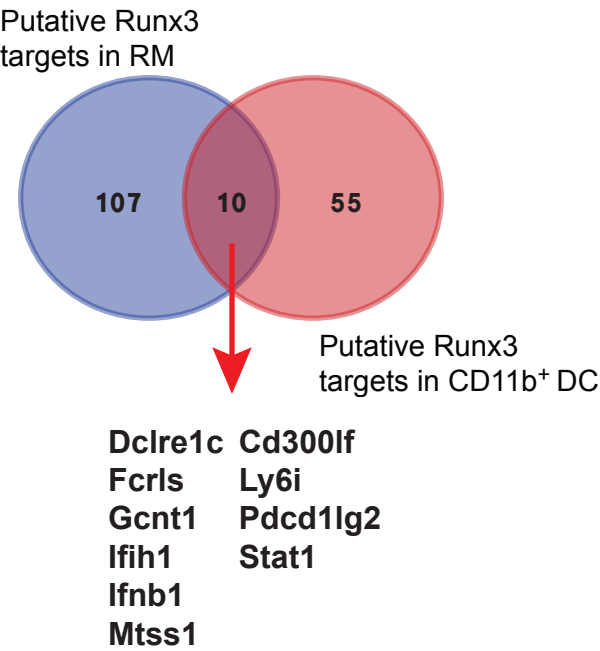

c

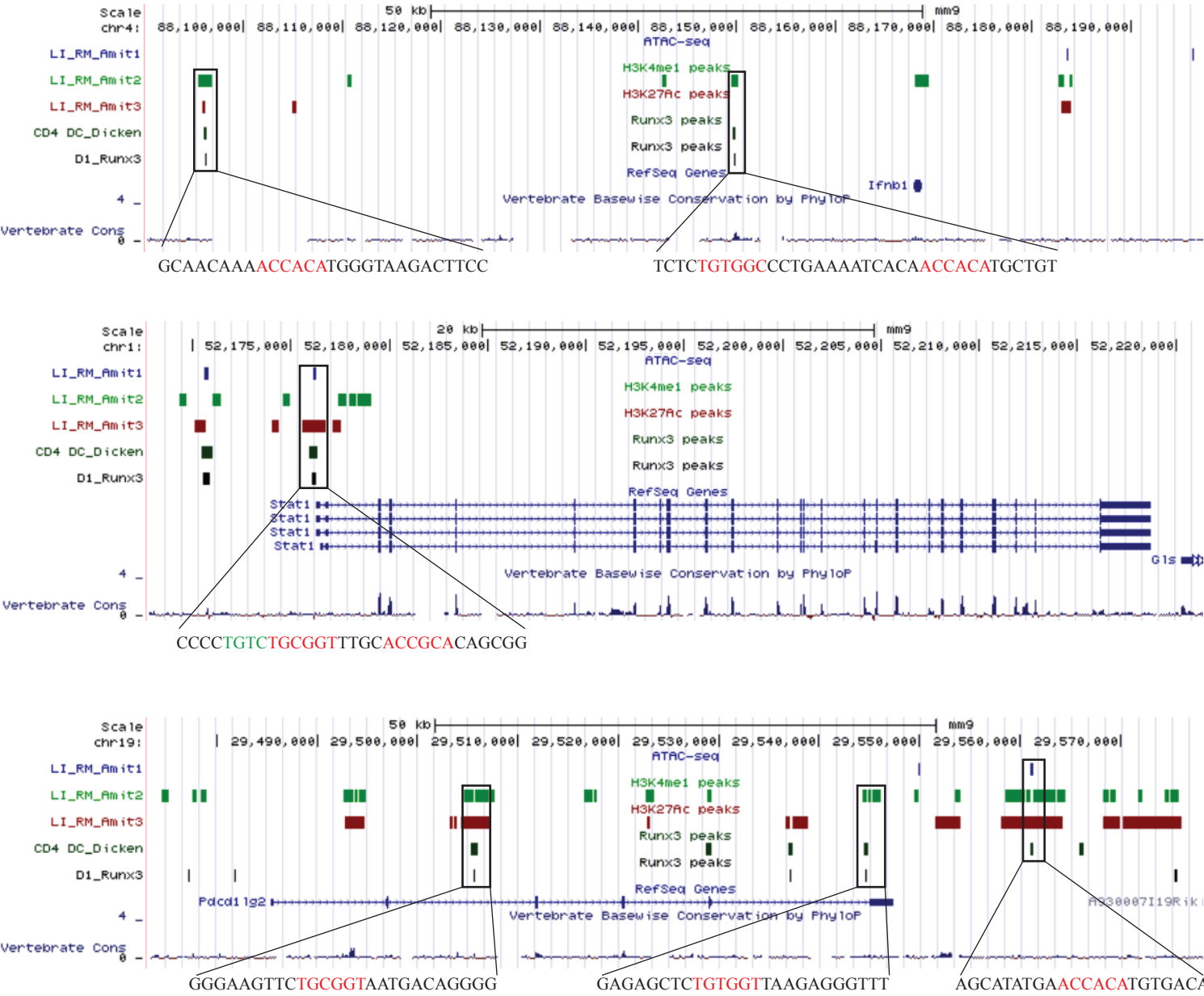

Supplement: S6 Fig — (a) Venn diagram depicting cross-analysis of DEGs in colonic Runx3Δ CD11b+ DC with genes harboring Runx3 peaks in D1 cells and splenic CD4+ DC. (b) Venn diagram depicting cross-analysis of Runx3 target genes in CD11b+ DC and Runx3 target genes in colonic RM. (c) UCSC genome browser display (mm9) of D1 cells and splenic CD4+ DC Runx3 occupied regions in three high-confidence DEGs (Ifnb1, Stat1 and Pdcd1lg2) common to colonic CD11b+ DC and RM. (PDF) [file pone.0233044.s006.pdf]

Thymocytes  
CD8<sup>+</sup> WT  
CD8<sup>+</sup> LCK-Cre

75 —  
50 —  
37 —  
25 —

Runx1  
Runx3

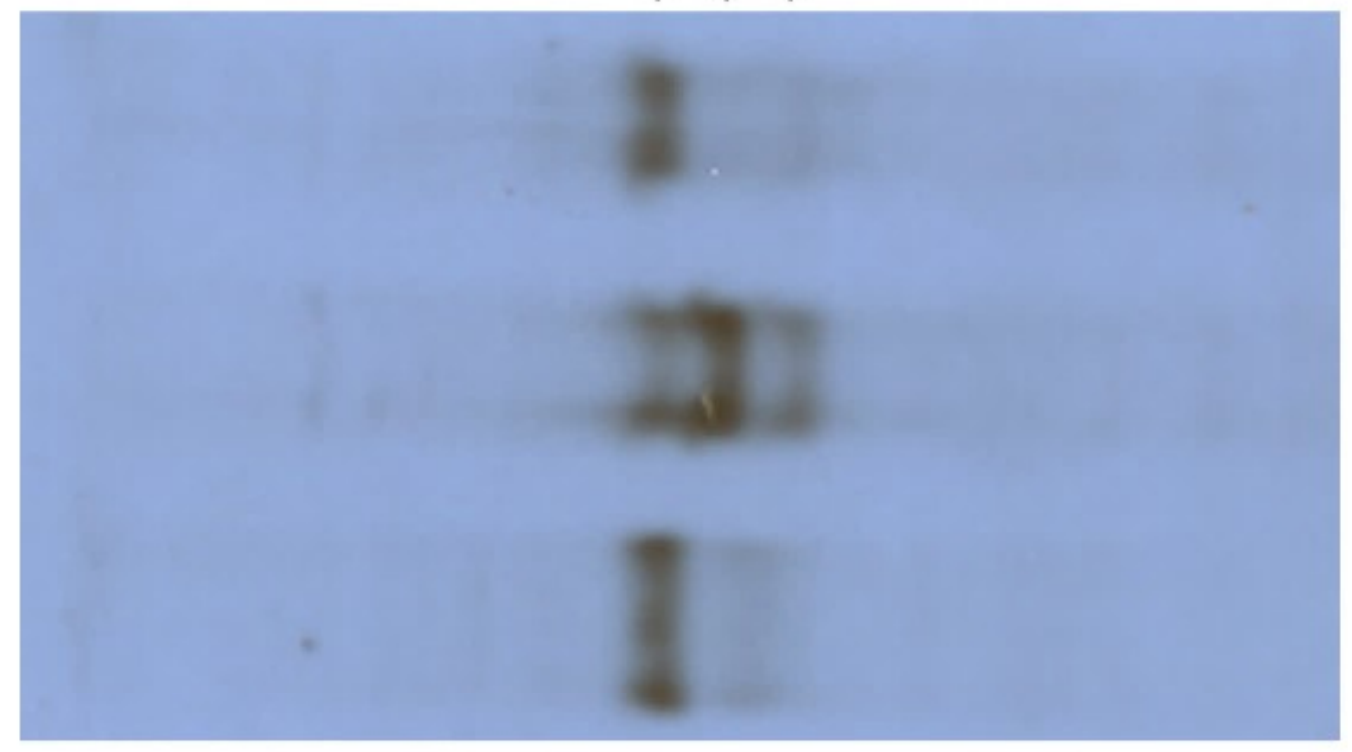

Emerin

Supplement: S7 Fig — Protein extract from mouse thymocytes or FACS-sorted splenic CD8+ T cells from WT or Runx3fl/fl:Lck-Cre mice were loaded (10 μg/lane) on SDS-polyacrylamide gel and separated by electrphoresis. Proteins were blotted onto nitrocellulose membrane and blots reacted sequentially with in house anti-Runx1, anti-Runx3 and anti-Emerin antibodies, followed by peoxidase-conjugated anti-rabbit IgG secondary antibody. Signals were developed using the ECL kit (Amersham Pharmacia) and detected by exposure to an x-ray film. (PDF) [file pone.0233044.s007.pdf]
